# The multifaceted role of autophagy and mitophagy in cardiovascular health and disease

**DOI:** 10.1080/27694127.2025.2572511

**Published:** 2025-10-16

**Authors:** Mireia Nàger, Mauro Calvoli, Kenneth B. Larsen, Asa B. Birgisdottir

**Affiliations:** a Department of Clinical Medicine, UiT-The Arctic University of Norway, Tromsø, Norway; b Department of Medical Biology, UiT-The Arctic University of Norway, Tromsø, Norway; c Division of Cardiothoracic and Respiratory Medicine, University Hospital of North Norway, Tromsø, Norway

**Keywords:** Aging, atherosclerosis, development, genetic mouse models, heart failure, myocardial infarction

## Abstract

The cardiovascular system, consisting of the heart and blood vessels, ensures delivery of oxygen and nutrient-rich blood throughout the whole body. The major cell types include cardiomyocytes, endothelial cells, and vascular smooth muscle cells. Dramatic consequences, sometimes with a deadly outcome, may arise when the activity of cardiovascular cells is compromised. The cardiomyocytes are terminally differentiated cells and thus do not normally regenerate. To sustain the high energy demand of the beating heart, the cardiomyocytes contain a high amount of energy producing mitochondria. Adaptation to metabolic demands is an integral part of cellular homeostasis and involves autophagy. Autophagy is an evolutionary conserved intracellular degradation pathway of cellular constituents. Mitophagy refers to selective degradation of damaged, and thus potentially harmful, mitochondria through autophagy. Both autophagy and mitophagy are widely implicated in physiological and pathological processes within cardiovascular cells. In this review, we highlight studies applying genetic modifications in mouse models to reveal the impact of autophagy and mitophagy on cardiovascular health and disease.

## Introduction

Autophagy is an evolutionarily conserved catabolic intracellular process culminating in lysosomal degradation of cellular constituents, including protein aggregates and damaged or surplus organelles such as mitochondria (then specifically termed mitophagy). Under basal conditions, autophagy functions constitutively^[1]^ but can be induced during stress conditions such as nutrient deprivation or hypoxia^[2]^. As an integral part of cellular homeostasis, autophagy is widely implicated in physiological processes such as development and aging as well as pathophysiological processes, including cardiovascular disease^[3–5]^. The cardiovascular system comprises the heart, arteries, veins, and capillaries and the major cell types include cardiomyocytes, vascular smooth muscle cells, endothelial cells, and immune cells^[6]^. The cycles of contractions and relaxations of the cardiomyocytes in the heart pump oxygen and nutrient-rich blood through the vessels, providing blood supply throughout the body. The heart needs vast amount of energy in the form of adenosine triphosphate (ATP) to sustain its continuous pumping function^[7]^. Through their oxidative phosphorylation activity, mitochondria are the major ATP producing organelles in cells. Cardiomyocytes have a very high abundance of mitochondria and mitochondrial function/dysfunction is therefore of essence in both pathogenesis and cellular compensation of various cardiovascular disorders.

**Figure 1.**
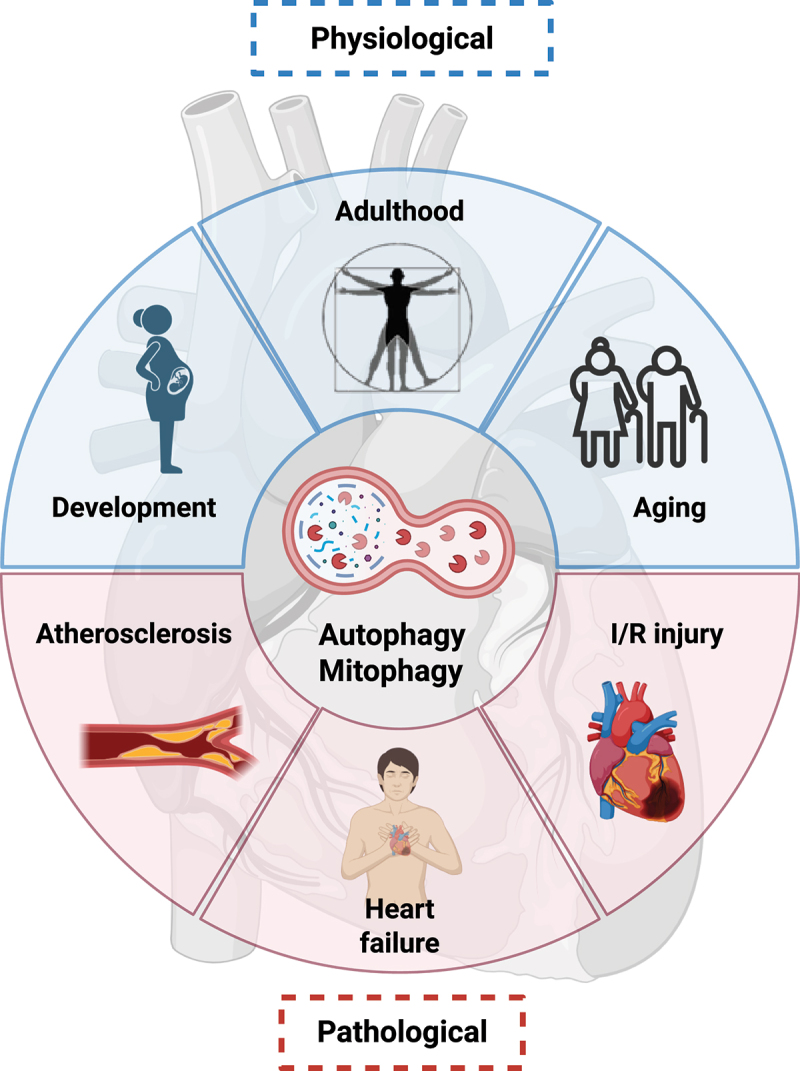
Schematic illustration of how autophagy and mitophagy impact on various conditions, including physiological states (development, adulthood and aging) as well as pathological conditions (atherosclerosis, heart failure and ischemia/reperfusion injury). Created in BioRender. Nàger, M. (2025). https://BioRender.com/axfu5zm.

In this review, we discuss the physiological role of autophagy and mitophagy in the cardiovascular system and how these mechanisms are important in cardiovascular disease development (Figure 1). We primarily focus on reported genetic interventions in animal models (mostly mice) with an impact on autophagy or mitophagy, resulting in a beneficial or disruptive effect on cardiovascular homeostasis (Table 1). To limit the scope of the review, we do not highlight animal studies that center on investigating pharmacological modulations of autophagy or mitophagy.

**Table 1.** Genetic Interventions in Mouse Models Correlated with Autophagy or Mitophagy in Cardiovascular Health and Disease.

Gene	Genetic modification/ /Cell type	Condition	Effect on Autophagy or mitophagy	Autophagy/mitophagy assessment; Flux ±	Cardiac phenotype	Ref
*Acacb*	Conditional cardiomyocyte-specific deletion	Disease (HFpEF)	Induced mitophagy	**Mitophagy flux**: Mito-Keima (cardiac tissue): increased number of acidic puncta WB (cardiac tissue) + CQ: increased MAP1LC3B-II	Attenuated HF phenotype, improved cardiac function and hypertrophy	Yoshii et al.^[247]^
*Adipoq*	Whole-body deletion	Disease (MI)	Impaired autophagy	WB (cardiac tissue): decreased pULK1-S555, increased p-PRKAA2-T172 WB (infarct region tissue homogenate): increased BECN1, MAP1LC3B-II, SQSTM1	Exacerbated apoptosis and cardiac dysfunction with increased fibrosis upon MI	Sung et al.^[200]^
*APOM*	Overexpression (human APOM)	Disease (DIC)	Preserved autophagy	**Autophagy flux**: IHC (cardiac tissue) + DOX + CQ: increased MAP1LC3B, SQSTM1	Unaltered LV mass, heart weight, and LV ejection fraction, presence of fibrosis	Guo et al.^[258]^
*Atg3*	Conditional cardiomyocyte-specific deletion	Baseline	Impaired autophagy	WB (cardiac tissue): decreased MAP1LC3B-II, increased SQSTM1	Hypertrophy and cardiac dysfunction with fibrosis, increased apoptosis, reduced life-span and reduced mitochondrial metabolism and biogenesis	Zhang et al.^[89]^
*Atg5*	Whole-body overexpression	Baseline	Induced autophagy	WB (cardiac tissue): increased ATG5, ATG12-ATG5, MAP1LC3B-II, decreased SQSTM1	Increased lifespan, normal age-dependent fibrosis	Pyo et al.^[143]^
*Atg5*	Conditional cardiomyocyte-specific deletion (E7.5 - E8)	Baseline	Impaired autophagy	WB (cardiac tissue): increased SQSTM1, poly-Ub	Premature death, cardiac abnormalities leading to HF, collapsed mitochondria and reduced mitochondrial respiration	Taneike et al.^[133]^
*Atg5*	Conditional cardiomyocyte-specific deletion	Baseline	Impaired autophagy	WB (isolated CMs): suppressed MAP1LC3B-I to II conversion, increased SQSTM1	Left ventricular dilation, severe contractility impairment, hypertrophy, impaired Ca^2+^ cycling, HF	Nakai et al.^[84]^
*Atg5*	Macrophage-specific heterozygous deletion	Disease (Ang II induced HF)	Impaired Ang II-induced mitophagy	WB (macrophages): decreased MAP1LC3B-II IF (macrophages): decreased MAP1LC3B puncta TEM + Ang II: decreased number of autophagic vacuoles	Enhanced cardiac damage after Ang II treatment, increased inflammatory cell infiltration and ROS production	Zhao et al.^[226]^
*Atg5*	Macrophage-specific deletion	Disease (atherosclerosis)	Impaired autophagy	WB (macrophages): MAP1LC3B-II absent in KO and lipid loaded KO **Autophagy flux**: WB (macrophages) + BafA_1_: no MAP1LC3B-II	Enhanced atherogenic plaque progression and impaired delivery of lipid droplets to lysosomes	Ouimet et al.^[179]^
*Atg5*	Macrophage-specific deletion	Disease (atherosclerosis)	Impaired autophagy	IF (atherosclerotic tissue): increased EGFP-MAP1LC3B puncta and SQSTM1 signal after western diet	Normal body weight, plasma lipids, lipoproteins, and plasma insulin, higher lesion and necrotic area in aortic root	Liao et al.^[180]^
*Atg5*	Macrophage-specific deletion in *apoe*^*-/-*^ background	Disease (atherosclerosis)	Impaired autophagy	WB (whole aorta lysates): increased SQSTM1 in control and after western diet	Increased aortic lesions and plaque formation after western diet with macrophagic inflammasome hyperactivation	Razani et al.^[181]^
*Atg5*	Macrophage-specific deletion in *apoe*^*-/-*^ background	Disease (atherosclerosis)	Impaired autophagy	WB (macrophages): increased MAP1LC3B IF (macrophages) + leucine: increased MAP1LC3B puncta **Autophagy flux**: Mito-Keima + CCCP: unchanged signal	Elevated atherosclerosis with highly complex lesions rich in apoptotic macrophages and necrotic cores	Zhang et al.^[188]^
*Atg5*	EC-specific deletion	Baseline	Impaired autophagy	WB (isolated aortic EC): reduced MAP1LC3B-II IF: increased MAP1LC3B puncta	Disturbed endothelial alignment during high shear stress and increased apoptosis and senescence with HFD.	Vion et al.^[110]^
*Atg5*	EC-specific deletion in *apoe*^*-/-*^ background	Disease (atherosclerosis)	Impaired autophagy	WB (isolated aortic EC): decreased MAP1LC3B-II	High formation of plaques	Vion et al.^[110]^
*Atg7*	Cardiomyocyte-specific overexpression	Baseline	Induced autophagy	EGFP-MAP1LC3 (cardiac tissue): increased puncta TEM: increase in autophagic structures **Autophagy flux**: WB (cardiac tissue) + CQ: no changes in SQSTM1, increased MAP1LC3B-II	Normal function and morphology of hearts.	Bhuiyanet al.^[86]^
*Atg7*	Cardiomyocyte-specific overexpression in CRYAB^R120G^ background	Disease (desmin-related cardiomyopathy-HF)	Induced autophagy	WB (cardiac tissue): increased SQSTM1 EGFP-MAP1LC3B : increased puncta **Autophagy flux**: WB (cardiac tissue) + CQ: increased MAP1LC3B-II, no changes in SQSTM1 mRFP-EGFP-MAP1LC3B: increased number of autophagosomes and autolysosomes	Delayed hypertrophy and cardiac fibrosis development, extended lifespan	Bhuiyan et al.^[86]^
*Atg7*	Conditional cardiomyocyte-specific deletion	Disease (I/R)	Impaired autophagy	WB (cardiac tissue): increased SQSTM1, decreased MAP1LC3B-II IF (cardiac tissue): reduced MAP1LC3B puncta; SQSTM1 does not colocalize with LAMP2	Severe contractile dysfunction and increased susceptibility to I/R injury	Li et al.^[205]^
*Atg7*	Cardiomyocyte-specific deletion	Disease (PO HF)	Impaired autophagy and mitophagy	WB (cardiac tissue): no MAP1LC3B-II TEM: Increased number of autophagosomes containing mitochondria after TAC **Mitophagy flux**: Mito-Keima (cardiac tissue) + TAC: decreased number of acidic puncta	Cardiac function not described	Nah et al.^[244]^
*Atg7*	EC-specific deletion	Baseline	Impaired autophagy	WB (isolated aortic EC): reduced MAP1LC3B-II IF: increased MAP1LC3B puncta	Slightly higher spleen and heart weights, disturbed endothelial alignment in high shear stress, increased senescence with HFD and in high-shear stress	Vion et al.^[110]^
*Atg7*	VSMC-specific deletion in *apoe*^*-/-*^ background	Disease (atherosclerosis)	Impaired autophagy	IF (carotid artery tissue): increased SQSTM1	Atherosclerotic plaque formation after tandem stenosis with autophagy deficiency-mediated instability and macroscopic hemorrhage	Masuyama et al.^[173]^
*Atg7*	VSMC-specific deletion in *apoe*^*-/-*^ background	Disease (atherosclerosis)	Impaired autophagy	WB (descending aorta): increased SQSTM1 in normal and western diet	Increased apoptosis and reduced survival with western diet, enhanced atherosclerotic changes with inflammation and outward arterial remodeling	Osonoi et al.^[126]^
*Atg7*	VSMC-specific deletion	Baseline	Impaired autophagy	WB (aortic segments): increased SQSTM1, decreased MAP1LC3B-II	Decreased aortic compliance, aortic extracellular matrix remodeling	De Munck et al.^[127]^
*Atg7*	VSMC-specific deletion	Baseline	Impaired autophagy	WB (aortic VSMC): increased SQSTM1, decreased MAP1LC3B-II	Accelerated stress-induced senescence, increased protection against oxidative stress-induced cell death, hypertrophy, increased migration capacity, increased collagen amount	Grootaert et al.^[148]^
*Becn1*	Cardiomyocyte-specific overexpression	Disease (HF)	Induced autophagy	GFP-MAP1LC3 (cardiac tissue): increased puncta after starvation and after sTAB	Impaired pathological cardiac remodeling after sTAB and pathological cell growth	Zhu et al.^[225]^
*Becn1*	Whole-body heterozygous deletion	Disease (HF)	Impaired autophagy	GFP-MAP1LC3 (cardiac tissue): decreased puncta after starvation and after sTAB	Reduced pathological decline in systolic performance after sTAB with no changes in compensatory hypertrophic growth	Zhu et al.^[225]^
*Becn1*	Whole-body heterozygous deletion	Disease (MI)	Impaired autophagy	WB (cardiac tissue): decreased MAP1LC3B-II	Increased myocardial infarct size	Xu et al.^[68]^
*Becn1*	Whole-body mutant (BECN^F121A^)	Baseline	Induced autophagy	WB (cardiac tissue): increased MAP1LC3B-II, decreased SQSTM1 **Autophagy flux**: GFP-MAP1LC3B (cardiac tissue) + CQ: increased puncta	Increased lifespan	Fernández et al.^[144]^
*Becn1*	Whole-body mutant (BECN^F121A^)	Baseline (Aging)	Induced autophagy	**Autophagy flux**: GFP-MAP1LC3B (cardiac tissue) + CQ: increased puncta WB (cardiac tissue) + CQ: decreased SQSTM1	Cardiac function not described	Sebti et al.^[145]^
*Becn1*	Whole-body heterozygous deletion in CRYAB^R120G^ background	Disease (desmin related cardiomyopathy HF)	Impaired autophagy	WB (cardiac tissue): increased Ub	Severe structural abnormalities, fibrosis, and accelerated HF progression	Tannous et al.^[238]^
*Becn1*	Whole body heterozygous deletion	Disease (Chronic DIC)	Restored autophagy	**Autophagy flux (heart)**: WB + BafA1 + DOX: increased MAP1LC3B-I to -II conversion, increased SQSTM1 RFP-GFP-MAP1LC3B + DOX: decreased autolysosomes	Preserved systolic performance, reduced ventricular dilation and fibrosis after long-term exposure to DOX	Li et al.^[253]^
*Becn1*	Whole body overexpression	Disease (DIC)	Impaired autophagy	**Autophagy flux**: WB (cardiac tissue) + BafA_1_ + DOX: no change in MAP1LC3B-II and SQSTM1 RFP-GFP-MAP1LC3B (cardiac tissue) + DOX: increased autolysosomes	Decline in systolic function, induced ventricular dilation and fibrosis	Li et al.^[253]^
*Bnip3*	Whole body deletion	Disease (DIC)	Not stated	TEM (cardiac tissue) + DOX: normalized autophagosomes	Lower mortality rate, almost normalized cardiac function	Dhingra et al.^[263]^
*Cav1, Ldlr*	Whole-body deletion in *ldlr*^*-/-*^background	Baseline	Induced autophagy	TEM (aortic sections): increased number of autophagic vacuoles **Autophagy flux** IF (aortic sections) + CQ: increased MAP1LC3B, increased SQSTM1	Increased atheroprotection by reduced endothelial cell activation.	Zhang et al.^[167]^
*Ccpg1*	Hypomorphic (loss of function) in mice with ER-phagy reporter	Disease (DIC)	Decreased ER-phagy in response to dox	**ER-phagy flux**: Abolished increase in acidic puncta of ER-phagy reporter after DOX treatment	Enhances ER-stress and apoptosis induced by dox.	Nakagama et al.^[260]^
*CK2a*	Cardiomyocyte-specific deletion	Disease (I/R)	Not stated for mouse tissue (impaired mitophagy in cell culture)	WB (cardiac tissue): decreased p-FUNDC1Ser13, increased mitochondrial MAP1LC3B-II, decreased SQSTM1	Restored heart function after I/R injury	Zhou et al.^[213]^
*Dnase2*	Cardiomyocyte-specific deletion	Disease (PO HF)	Impaired autophagy	IF (cardiac tissue): increased colocalization of picogreen/EdU (mtDNA) with LAMP2a and MAP1LC3B	Decreased survival rate and fibrosis after TAC, cardiac dysfunction with immune cell infiltration and inflammation after TAC	Oka et al.^[231]^
*Dnm1l*	Conditional cardiomyocyte-specific deletion	Baseline	Impaired autophagy	WB (cardiac tissue): decreased MAP1LC3B-II, increased SQSTM1 **Autophagy flux**: WB (cardiac tissue) + CQ: no changes in MAP1LC3B and SQSTM1 mRFP-GFP-LC3: no increase in red-only puncta	Premature death, hypertrophy with fibrosis, elongated/enlarged mitochondria with decreased ATP production	Ikeda et al.^[107]^
*Dnm1l*	Conditional cardiomyocyte-specific deletion	Disease (HFD induced HF)	Impaired autophagy and mitophagy	GFP-MAP1LC3B (cardiac tissue) + HFD: decreased number of puncta **Autophagy flux**: GFP-MAP1LC3B + CQ: increased number of puncta in HFD, increased MAP1LC3B-II in normal or HFD + CQ **Mitophagy flux**: Mito-Keima + HFD: decreased number of acidic puncta; no presence of YFP-Rab9 puncta after HFD	Exacerbation of cardiac dysfunction and reduced viability with HFD, reduced LV fractional shortening	Tong et al.^[72]^
*Dnm1l*	Conditional cardiomyocyte-specific heterozygous deletion	Disease (I/R)	Impaired autophagy	WB (cardiac tissue) + I/R: reduced MAP1LC3B-II, SQSTM1 TEM: reduced level of autophagosomes containing mitochondria	Larger infarct area, increased mitochondrial fission after I/R	Ikeda et al.^[107]^
*Ficd*	Whole body deletion	Disease (HF)	Induced autophagy Induced ER-phagy	**Autophagy flux**: WB + BafA_1_: increased MAP1LC3B-II, SQSTM1 WB: increased CathepsinL **ER-phagy flux**: WB + BafA_1_ + ER stress: accumulated RETREG1 (ER-phagy receptor)	Abolished cardiac hypertrophy, fibrosis and HF after TAC. Increased ER structures in autophagosomes (TEM)	Lacy et al.^[233]^
*Fundc1*	Whole-body deletion	Baseline	Impaired mitophagy	WB (cardiac tissue): no difference in MAP1LC3B-II, increased TOMM20	Decline in left ventricular systolic function	Xu et al.^[68]^
*Fundc1*	Deletion	Disease (MI)	Impaired mitophagy	WB (cardiac tissue): no changes in MAP1LC3B-II, increased TOMM20	Increased myocardial infarct size	Xu et al.^[68]^
*Fundc1*	Whole-body overexpression	Baseline	Induced autophagy and mitophagy	WB (cardiac tissue): increased MAP1LC3B-II, increased MAP1LC3B-II in mitochondrial fraction, decreased TOMM20	Decline in cardiac function with LV dilation	Xu et al.^[68]^
*Fundc1*	Whole-body overexpression	Disease (MI)	Induced autophagy and mitophagy	WB (cardiac tissue): increased MAP1LC3B-II, increased MAP1LC3B-II in mitochondrial fraction, decreased TOMM20	Alleviated cardiac dysfunction after MI, improved mitochondrial respiratory function	Xu et al.^[68]^
*Fundc1*	Cardiomyocyte-specific deletion	Baseline and disease (HF)	Induced mitophagy	TEM: elongated mitochondria, and accumulation of dysfunctional mitochondria **Autophagy flux**: Mito-Keima (cardiac tissue): increased number of acidic puncta	Apoptosis and cardiac injury with fibrosis	Wu et al.^[102]^
*Fundc1*	Cardiomyocyte-specific overexpression	Disease (DIC)	Impaired autophagy	**Autophagy flux**: WB (cardiac tissue) + DOX + BafA_1_: increased MAP1LC3B-II IF (cardiac tissue) + DOX + BafA_1_: increased MAP1LC3B puncta	Improved cardiac function with reduced fibrosis	He et al.^[265]^
*Fundc1, Becn1*	BECN1 (±) Overexpression Fundc1	Disease (MI)	Same increased autophagy and mitophagy levels as *Fundc1* TG mice	WB (cardiac tissue): increased MAP1LC3B-II	Alleviated cardiac function from acute MI compared with *Beclin1*^±^ mice	Xu et al.^[68]^
*Fyco1*	Whole-body deletion	Disease (PO HF)	Impaired autophagy	WB (cardiac tissue): no change in MAP1LC3B-I and II in fed and starved mice WB (cardiac tissue) + TAC: no increase in MAP1LC3B-II **Autophagy flux**: WB (cardiac tissue) + CQ + starvation: no increase in MAP1LC3B-II	Normal phenotype in normal conditions with development of contractile dysfunction upon starvation or biomechanical stress (PO)	Kuhn et al.^[228]^
*FYCO1*	Whole-body overexpression (human FYCO1)	Disease (PO HF)	Induced autophagy	GFP-MAP1LC3B (cardiac tissue): increased puncta TEM: increased number of autophagosomes **Autophagy flux**: GFP-MAP1LC3B (cardiac tissue) + CQ + starvation: further increased puncta WB (cardiac tissue): increased MAP1LC3B-II WB (cardiac tissue) + CQ + starvation: increased MAP1LC3B-II	Mild cardiac hypertrophy and rescued PO-induced contractile dysfunction	Kuhn et al.^[228]^
*Gsk3b*	Cardiomyocyte-specific overexpression (dominant negative GSK-3b)	I/R	Impaired autophagy	WB (cardiac tissue): increased SQSTM1 GFP-MAP1LC3B (cardiac tissue): decreased puncta	Increased infarct size after ischemia (2 h), decreased infarct size after I/R	Zhai et al.^[203]^
*Lamp2*	Whole-body deletion	Baseline (Danon disease)	Not stated	TEM: accumulation of autophagic vacuoles filled with polymorphic content	Increased mortality	Tanaka et al.^[91]^
*Lamp2*	Whole-body expression of a deletion mutant (exon 6 deleted)	Baseline (Danon disease)	Impaired autophagy	WB (cardiac tissue): increased MAP1LC3B-II TEM (cardiac tissue): increased autophagosomes	Hypertrophy and fibrosis, abnormal relaxation and altered calcium homeostasis in myocytes	Alcalai et al.^[92]^
*Mfn1, Mfn2, Dnm1l*	Conditional cardiomyocyte-specific deletion	Baseline	Impaired mitophagy	WB (cardiac mitochondrial fraction): reduced Ub, MAP1LC3B-II, SQSTM1	Concentric cardiac hypertrophy, increased LV mass, decreased pump performance	Song et al.^[109]^
*Mfn2*	Conditional cardiomyocyte-specific mutant overexpression (MFN2^AA^) and *Prkn* deletion	Baseline	Impaired mitophagy	WB (cardiac mitochondrial fraction): decreased SQSTM1, PRKN	Progressive perinatal cardiomyopathy with HF and premature death	Gong et al.^[100]^
*Mfn2*	Conditional cardiomyocyte-specific mutant overexpression (MFN2^EE^) and *Prkn* deletion	Baseline	Induced mitophagy	WB (cardiac mitochondrial fraction): increased SQSTM1, PRKN	Normal phenotype	Gong et al.^[100]^
miRNA-199a	Cardiomyocyte-specific overexpression	Baseline	Impaired autophagy	WB (cardiac tissue): increased SQSTM1, decreased MAP1LC3B-II TEM (cardiac tissue): decreased autophagic vacuoles **Autophagy flux**: WB + CQ: reduced MAP1LC3-II, increased SQSTM1	Early cardiac hypertrophy, increased fibrosis and HF, premature death	Li et al.^[97]^
*miRNA-212 and miRNA-132*	Whole-body deletion of miRNA-212 and miRNA-132	Baseline and disease (PO HF)	Induced autophagy	WB (cardiac tissue): increased MAP1LC3B-II, decreased SQSTM1	Smaller hearts, normal cardiac function parameters, protection from PO-induced HF	Ucar et al.^[96]^
miRNA-212 and miRNA-132	Cardiomyocyte-specific overexpression	Baseline	Impaired autophagy	WB (cardiac tissue): decreased MAP1LC3B-II, increased SQSTM1	Premature death, cardiac hypertrophy resulting in HF development	Ucar et al.^[96]^
*Mst1*	Cardiomyocyte-specific overexpression	Baseline	Impaired autophagy	WB (cardiac tissue): increased Ub, SQSTM1, decreased MAP1LC3B-II **Autophagy flux**: GFP-MAP1LC3B (cardiac tissue) + CQ: decreased puncta TEM (cardiac tissue): increased number of autophagosomes	Promoted cardiac dysfunction	Maejima et al.^[204]^
*Mst1*	Whole-body deletion	Disease (I/R)	Induced autophagy	WB (cardiac tissue) + MI: increased MAP1LC3B-II, decreased SQSTM1 GFP-MAP1LC3B (cardiac tissue) + MI: increased puncta TEM (cardiac tissue) + starvation: increased number of autophagosomes	Cardiac protection and improved remodeling after I/R	Maejima et al.^[204]^
*Mst1*	Cardiomyocyte-specific overexpression (dominant negative MST1)	Disease (I/R)	Induced autophagy	WB (cardiac tissue) + MI: increased MAP1LC3B-II, decreased SQSTM1 GFP-MAP1LC3 (cardiac tissue) + MI: increased puncta TEM (cardiac tissue) + starvation: increased number of autophagosomes	Cardiac protection and improved remodeling after I/R	Maejima et al.^[204]^
*Mtor*	Conditional cardiomyocyte-specific heterozygous deletion	Disease (MI)	Not stated	WB (cardiac tissue): increased MAP1LC3B-II in control and after HFD	Reduction in infarct size after MI	Sciarretta et al.^[196]^
*Prkaa2*	Whole-body deletion	Disease (PO HF)	Impaired mitophagy	IF (cardiac tissue): increased SQSTM1 TEM (cardiac tissue): no autophagosomes with mitochondria WB (cardiac mitochondrial fraction): no PRKN, p-PARKN S65, SQSTM1, PINK1	Aggravated early HF induced by TAC with reduced ejection fraction and increased heart weight	Wang et al.^[241]^
*Prkn*	Whole-body deletion	Baseline	No change	WB: no differences in MPA1LC3B-II levels	Normal cardiac function	Kubli et al.^[99]^
*Prkn*	Whole-body deletion	Disease (MI)	Impaired mitophagy	WB (cardiac infarct border zone tissue): reduced MAP1LC3B-II	Increased mortality and reduced remodeling after MI, damaged mitochondria	Kubli et al.^[99]^
*Prkn*	Whole body deletion	Disease (Cardiac aging and DIC)	Impaired mitophagy	TEM + DOX: reduced number of mitochondria in autophagosomes	Lower contractility	Hoshino et al.^[152]^
*Prkn, Dnm1l*	Conditional cardiomyocyte-specific deletion	Disease (Cardiomyopathy)	Impaired mitophagy compared to *dnml1* KO	WB (mitochondrial fractions from cardiac tissue): reduced SQSTM1, Ub, MAP1LC3B-II TEM (cardiac tissue): increased abundance of mitochondria	Increased survival, improved contractile function, protection against ventricular remodeling, reduced fibrosis	Song et al.^[101]^
*Rab9a*	Whole-body mutant overexpression(RAB9A^S179A^) in HFD mice	Disease	Impaired mitophagy	**Mitophagy flux**: Mito-Keima (cardiac tissue) + HFD: decreased number of acidic puncta	Exacerbated cardiac hypertrophy and increased fibrosis, increased lipid accumulation	Tong et al.^[246]^
*Rheb*	Conditional cardiomyocyte-specific overexpression	Disease (MI)	Impaired autophagy	WB + ischemia + rapamycin: increased MAP1LC3B-II, decreased SQSTM1	Increased apoptosis and necrosis leading to increased MI size after ischemia	Sciarretta et al.^[196]^
*Rnf7*	Conditional cardiomyocyte-specific deletion	Disease (HF)	Impaired mitophagy	WB (isolated mitochondria from isolated CMs): decreased SQSTM1, MAP1LC3B-II IF (mitophagy dye): reduction in mitophagy vesicles	Hypertrophy and impaired cardiac contractility, dilated cardiomyopathy and HF	Wang et al.^[104]^
*Rptor*	Macrophage-specific deletion in apoe^−/−^ background	Disease (atherosclerosis)	Induced mitophagy	IF (isolated macrophages) + leucine: increased MAP1LC3B puncta **Autophagy flux**: WB + BafA_1_: increased MAP1LC3B-II Mito-Keima + CCCP: loss of acidic puncta	Decreased atherosclerotic lesion formation after western diet with reduced macrophages, apoptotic cells, and necrotic core area in plaques	Zhang et al.^[188]^
*Rubcn*	Cardiomyocyte-specific deletion	I/R	Induced autophagy	TEM: increased number of autolysosomes and enlarged autophagosomes **Autophagy flux**: WB (cardiac tissue lysate from 30 min I/24 h R) + CQ: increased MAP1LC3B-II	Attenuated I/R damage, reduced infarct size and autosis in the heart tissue	Nah et al.^[209]^
*Rubcn*	Whole body deletion	Disease (DIC)	Induced autophagy and mitophagy	WB (cardiac tissue) + DOX: attenuated increase in MAP1LC3B-II and SQSTM1, attenuated reduction in PRKN in whole tissue but increase in mitochondria fraction and decrease in cytoplasmic fraction **Autophagy flux**: WB (cardiac tissue) + DOX + CQ: increased MAP1LC3B-II, SQSTM1	Improved survival, reduced fibrosis, unaltered cardiac function, attenuated reduction in LV thickness	Liu et al.^[255]^
*Slc39a7*	Conditional cardiomyocyte-specific deletion	Disease	Induced mitophagy	WB (cardiac mitochondrial fraction): Decreased TOMM20, TOMM22, citrate synthase, increased MAP1LC3B-II, PRKN, PINK1 **Autophagy flux**: Mito-QC: increase in acidic puncta both at baseline and after I/R	Reduced ROS production and reduced infarct area upon I/R injury	Zhang et al.^[214]^
*Tfeb*	Macrophage-specific overexpression	Baseline	Induced autophagy	**Autophagy flux**: WB (isolated macrophages) + BafA_1_: increased MAP1LC3B-II Live imaging of GFP-MAP1LC3B (isolated macrophages) + BafA_1_: increased green area	Atheroprotection in the whole aorta, 50% reduction in lesion area	Sergin et al.^[183]^
*Tfeb*	Macrophage-specific overexpression in apoe^−/−^ background	Disease (atherosclerosis)	Induced autophagy	IF (isolated macrophages) + western diet: increased SQSTM1, MAP1LC3B-II	Reduced development of atherogenic plaque and inflammation	Sergin et al.^[183]^
*Tfeb, Atg5*	Macrophage-specific overexpression (TFEB) and deletion (*Atg5*) in apoe^−/−^ background	Disease (atherosclerosis)	Impaired autophagy	WB (isolated macrophages): decreased MAP1LC3B-II	Loss of protection from atherosclerosis	Sergin et al.^[183]^
*Tfrc*	Cardiomyocyte-specific deletion	Baseline	Impaired mitophagy	WB (cardiac tissue): decreased MAP1LC3B-II, increased SQSTM1	Death at P11, hypertrophy, increased heart weight, LV dilation, decreased fractional shortening, enlarged CM (measurements done on P10 mice)	Xu et al.^[94]^
*Tlr9*	Whole body deletion	Disease (DIC)	Enhanced autophagy	WB (cardiac tissue) + DOX: p-ULK1, increased MAP1LC3B-II	Attenuated myocardial atrophy and cardiac fibrosis	Guo et al.^[257]^
*Tp53*	Whole-body deletion	Baseline Disease (Cardiac aging and DIC)	Induced mitophagy	TEM (cardiac tissue): increased number of autophagosomes with mitochondria, decreased number of abnormal mitocondria WB (cardiac mitochondrial fraction) + CCCP: increased PRKN, decreased MFN1 TEM + DOX: increased number of mitochondria in autophagosomes	Increased cardiac functionality compared to WT DOX: higher contractility	Hoshino et al.^[152]^
*Tp53, Prkn*	Double whole-body deletion	Disease (DIC)	Impaired mitophagy	TEM (cardiac tissue): decreased number of autophagosomes with mitochondria, increased number of abnormal mitochondria	Decreased cardiac functionality compared to WT	Hoshino et al.^[152]^
*Tsc2*	Whole-body mutant overexpression (TSC2^S1365A/WT^ or TSC2^S1365A/S1365A^)	Disease (PO HF)	Impaired autophagy after PO	WB (cardiac tissue): increased SQSTM1, decreased MAP1LC3B-II	Exacerbated hypertrophy and mortality from PO	Ranek et al.^[227]^
*Tsc2*	Whole-body mutant overexpression (TSC2^S1365E/WT^ or TSC2^S1365E/S1365^)	Disease (PO HF)	Induced autophagy after PO	WB (cardiac tissue): decreased SQSTM1, increased MAP1LC3B-II	Reduced hypertrophy and better heart function, reduced aggregation of myocardial proteins	Ranek et al.^[227]^
*Ulk1*	Cardiomyocyte-specific deletion	Disease (PO HF)	Impaired alternative mitophagy	WB (cardiac tissue) + TAC: increased MAP1LC3B-II, reduced SQSTM1 **Mitophagy flux**: Mito-Keima (cardiac tissue)+ TAC: decreased number of acidic puncta; increased signal after Tat-BECN1 TEM + TAC: increased number of autophagosomes containing mitochondria and autolysosomes	More severe cardiac dysfunction, hypertrophy, and fibrosis	Nah et al.^[244]^
*Ulk1*	Cardiomyocyte-specific deletion in HFD mice	Disease (HFD HF)	Impaired mitophagy	**Mitophagy flux**: Mito-Keima (cardiac tissue): decreased number of acidic puncta during normal diet and HFD TEM + HFD: decreased number of autophagosomes containing mitochondria	Exacerbated cardiac hypertrophy and increased myocardial fibrosis, aggravated mitochondrial disfunction after HFD	Tong et al.^[246]^
*Uvrag*	Whole body homozygous deletion	Disease (DIC)	Impaired autophagy	WB (cardiac tissue) + DOX: increased MAP1LC3B-II, SQSTM1, Ub IHC (cardiac tissue): increased MAP1LC3B puncta	Lower survival, development of fibrosis, decreased LV function	An et al.^[254]^

Baseline refers to no direct disease modeling in the mice. Abbreviations: *Acacb*: acetyl-CoA carboxylase beta; *Adipoq*: adiponectin, C1Q and collagen domain containing; AngII: angiotensin II; *ApoM*: apolipoprotein M; *Atg3*: autophagy related 3; *Atg5*: autophagy related 5; BafA1: bafilomycin A_1_; *Cav1*: caveolin 1; CCCP: Carbonyl Cyanide m-Chlorophenylhydrazone; *Ccpg1*: cell cycle progression 1; *CK2a*: casein kinase 2 alpha; CM: cardiomyocytes; CQ: chloroquine; DIC: doxorubicin-induced cardiotoxicity; *Dnase2*: deoxyribonuclease 2, lysosomal; DOX: doxorubicin; E7.5: embryonic day 7.5; EC: endothelial cell; *Ficd*: FIC domain protein adenylyltransferase; HF: heart failure; HFD: High-fat diet; HFpEF: heart failure with preserved ejection fraction; IF: immunofluorescence; IHC: immunohistochemistry; I/R: ischemia/reperfusion; KO: knockout; *Ldlr*: low density lipoprotein receptor; LV: left ventricle; LVFS: left ventricular fractional shortening; MI: myocardial infarction; *Mtor*: mechanistic target of rapamycin kinase; P11: postnatal day 11; PO: pressure overload; *Rnf7*: ring finger protein 7; ROS: reactive oxygen species; sTAB: severe thoracic aortic banding; TAC: transverse aortic constriction; TEM: transmission electron microscopy; TG: transgenic; *Tlr9*: toll like receptor 9; TOMM20: Translocase of the Outer Mitochondrial Membrane 2; *Tp53*: tumor protein p53; *Tsc2*: TSC complex subunit 2; Ub: ubiquitin; VSMC: vascular smooth muscle cell; WB: western blot.

## Molecular mechanisms of autophagy and mitophagy

**Figure 2.**
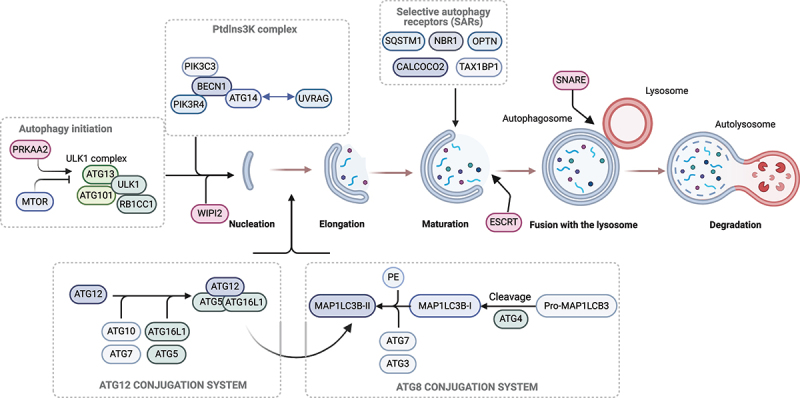
Overview of the autophagy pathway and the core autophagy machinery that orchestrates the pathway. Autophagy is regulated by the activity of the key metabolic sensors, MTOR and PRKAA2. While MTOR acts as an inhibitor, PRKAA2 functions as an activator of autophagy. Upon autophagy induction, the ULK1 complex (composed of ULK1, ATG13, ATG101 and RB1CC1) is activated, triggering the nucleation of the phagophore. This occurs through the phosphorylation of components of the Ptdlns3K complex I (consisting of PIK3C3, PIK3R4, BECN1 and ATG14) which directs the recruitment of the machinery necessary for autophagosome formation. Ptdlns3K-C1 promotes localized production of PtdIns3P on the forming phagophore membrane, leading to recruitment of WIPI2. WIPI2 binds to ATG16L1, facilitating the recruitment of ATG12–ATG5-ATG16L1 complex. This complex plays a critical role in the conjugation of MAP1LC3B to phosphatidylethanolamine (PE). Initially MAP1LC3B is cleaved by the protease ATG4 to form MAP1LC3B-I, which is subsequently conjugated to PE to generate MAP1LC3B-II. This process is mediated by enzymatic activity of the ATG7 and ATG3 proteins. MAP1LC3B-II is then incorporated into pre-autophagosomal and autophagosomal membranes, where MAP1LC3-II can interact with selective autophagy receptors (SQSTM1, NBR1, OPTN, CALCOCO2 and TAX1BP1). The ESCRT machinery contributes to the sealing of the autophagosomal membrane, resulting in the formation of a double-membrane vesicle called the autophagosome. The autophagosome matures and eventually fuses with the lysosome trough the action of SNARE complexes. In the resulting structure, called autolysosomes, the cargo is degraded, and its components are recycled. Created in BioRender. Nàger, M. (2025) https://BioRender.com/u7nt869.

Autophagy is initiated through the formation of double-membrane structures termed phagophores. The phagophores expand and close upon themselves, sequestering cellular cargo targeted for degradation within vesicles termed autophagosomes. These double-membrane vesicles ultimately fuse with acidic lysosomes, forming autolysosomes where the cargo is degraded and recycled^[8]^ (Figure 2). Chaperone-mediated autophagy^[9]^, microautophagy,^[10]^ and macroautophagy^[11]^ are three distinct forms of autophagy, characterized by different physiological functions and modes of cargo delivery to the lysosome. Macroautophagy, hereafter termed autophagy, is the form that appears to be mostly involved in both physiological and pathological mechanisms relevant for the cardiovascular system^[12]^. Nonselective, bulk autophagy refers to indiscriminate sequestration of cytosolic constituents, occurring in response to nutrient deprivation. In contrast, selective autophagy refers to autophagic degradation of specific, often harmful cargos such as damaged organelles (thereof the specific names such as mitophagy, ER (endoplasmic reticulum)-phagy, and lipo (lipid droplet)-phagy). Selective autophagy pathways serve as cellular quality control mechanisms and can be increased in response to cellular stress. Mitophagy is the most extensively studied selective autophagy pathway and mounting evidence indicates the importance of this process in the cardiovascular system^[13,14]^. The detailed molecular mechanisms of autophagy and mitophagy have been elegantly described elsewhere^[15,16]^. Hence, here we will only briefly describe the main mediators of autophagy and mitophagy.

### Autophagy

Autophagy is mainly orchestrated by the activity of autophagy-related (ATG) proteins. Initiation of autophagy requires the activation of the ULK1 (unc-51 like autophagy activating kinase 1) protein kinase complex consisting of RB1CC1/FIP200 (RB1 inducible coiled coil 1/200 kDa FAK family kinase-interacting protein), ATG13 (autophagy related 13) and ATG101 (autophagy related 101) along with ULK1. This complex subsequently recruits and phosphorylates downstream core-autophagy proteins such as components of the class III phosphatidylinositol 3-kinase complex I (PtdIns3K-C1), including ATG14 (autophagy related 14), BECN1 (Beclin1), and PIK3C3 (phosphatidylinositol 3-kinase catalytic subunit type 3)^[17]^. The PtdIns3K-C1 catalyzes the formation of phosphatidylinositol-3-phosphate (PtdIns3P) in the forming phagophore membrane, which then recruits effector proteins such as WIPI2 (WD repeat domain, phosphoinositide interacting 2) needed for membrane elongation^[18]^. Lipidation of the Atg8-family proteins such as MAP1LC3B (microtubule associated protein 1 light chain 3 beta) through conjugation to phosphatidylethanolamine (PE) for membrane insertion is a crucial step in autophagosome formation. Thus, lipidated MAP1LC3B (or LC3B-II) is a key molecular marker of autophagosomes and canonical autophagy^[19,20]^. As a multistep process, Atg8 or MAP1LC3B lipidation is facilitated by a chain of enzymatic activity involving the core autophagy proteins, the E1-like ATG7, the E2-like ATG3, and the E3-like ATG12–ATG5-ATG16L1 complex^[21–23]^ (Figure 2).

Selective autophagy in mammals involves de novo formation of autophagosomes on specific targets through cargo recognition by specialized receptor proteins. These are collectively termed selective autophagy receptors (SARs), subdivided into soluble and membrane bound selective autophagy receptors (SARs). The soluble selective autophagy receptors (SARs) include SQSTM1/p62 (sequestosome 1), TAX1BP1 (Tax1 binding protein 1), CALCOCO2/NDP52 (calcium binding and coiled-coil domain 2), NBR1 (NBR1 autophagy cargo receptor), OPTN (optineurin), and others. These receptors are responsible for bridging the cargo and the forming autophagosome by recognizing ubiquitin chains on the cargo and binding to Atg8-family proteins in the forming autophagosomal membrane via their LC3-interacting region (LIR) motif^[24,25]^. Phagophore closure, resulting in the formation of a double-membrane autophagosome, is carried out by the actions of the ESCRT (endosomal sorting complex required for transport) machinery^[26–29]^. Autophagosome maturation and lysosomal fusion involve the complex II variant of the PtIns3K complex (PtIns3K-C2), containing UVRAG (UV radiation resistance associated) instead of ATG14^[30,31]^. Finally, SNARE (soluble N-ethylmaleimide-sensitive factor attachment protein receptor) complexes are responsible for autophagosome fusion with lysosomes where the cytoplasmic cargo is degraded within acidic autolysosomes^[32,33]^ (Figure 2).

Of importance, the dynamic process of autophagosome formation, maturation, and degradation represents autophagic flux. Hence, it is necessary to monitor autophagic substrates over time to verify their degradation. Furthermore, an increase in abundance of autophagosomes can indicate an induction in autophagosome formation or a block in their degradation. Use of inhibitors that neutralize the lysosomal pH, such as the macrolide antibiotic bafilomycin A_1_, or inhibit autophagosome fusion with lysosomes, such as the anti-malaria drug chloroquine, can be used to distinguish if the flux is induced or inhibited^[34,35]^.

Adaptation to metabolic demands is the most fundamental role of autophagy. In nutrient-rich conditions, MTOR (mechanistic target of rapamycin) complex 1 (MTORC1) acts as the main negative regulator of autophagy in eukaryotic cells. Besides the evolutionary conserved kinase MTOR, the complex consists of several proteins, including RPTOR (regulatory associated protein of MTORC1)^[36]^. The active small GTPase RHEB (Ras homolog, mTORC1 binding) anchored at the lysosome outer surface activates MTORC1. This results in suppression of autophagy through inhibitory phosphorylations targeting the ULK1 complex^[37–39]^ the PtIns3K-C2^[40]^ as well as transcription factor EB (TFEB)^[41]^, a master regulator of autophagy and lysosomal genes^[42]^. The activity of MTOR can be inhibited by the lipophilic macrolide antibiotic rapamycin, a well-established inducer of autophagy^[43]^.

Low cellular energy supplies activate the PRKAA2/AMPK (protein kinase AMP-activated catalytic subunit alpha 2) which phosphorylates and negatively regulates MTORC1^[44,45]^. Furthermore, PRKAA2 binds and phosphorylates ULK1 and thus directly impacts on autophagy initiation. Recently, a new model on PRKAA2 and autophagy regulation was suggested, where instead of activating ULK1 by phosphorylation, PRKAA2 inhibits ULK1 and suppresses the immediate activation of autophagy during energy depletion. Notably, binding to PRKAA2 stabilizes essential autophagy components by preventing their degradation and thus contributes to preserving cellular homeostasis^[46,47]^.

### Mitophagy

**Figure 3.**
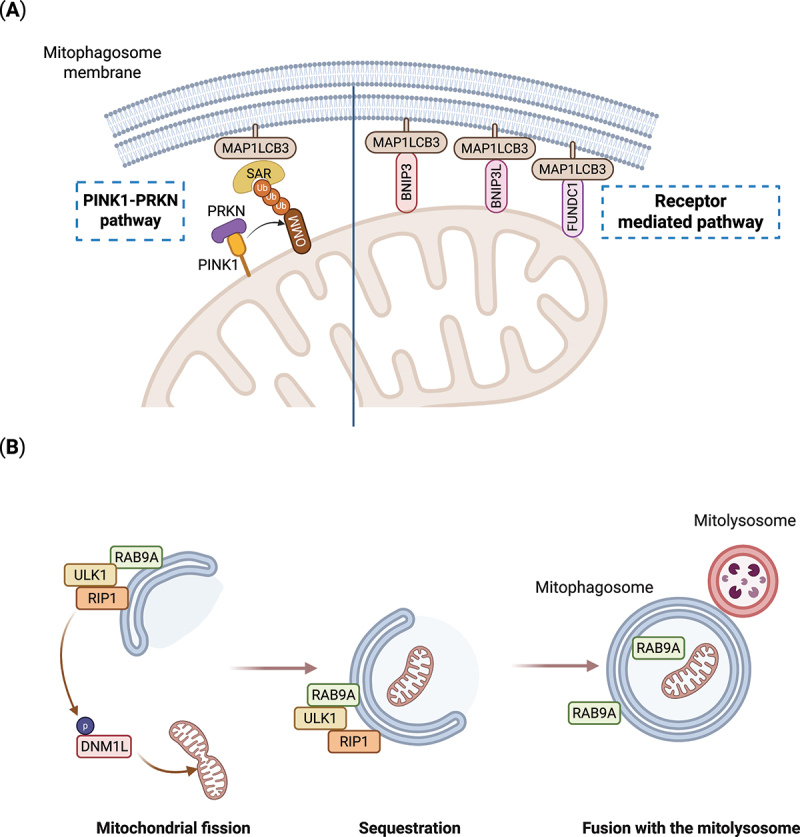
Overview of main mitophagy mechanisms. (A) PINK1-PRKN-mediated mitophagy (left) or receptor-mediated mitophagy (right). In the PINK1-PRKN pathway, PINK1 is stabilized on the outer mitochondrial membrane on depolarized mitochondria and subsequently recruits PRKN. PRKN, which is an E3 ubiquitin (ub) ligase, attaches ub to proteins (forming poly-ub chains) in the outer mitochondrial membrane (OMM). These poly-ub chains on the OMM are recognized by selective autophagy receptors (SARs), which then bind to Atg8-family proteins such as MAP1LC3B to initiate mitophagosome formation around damaged mitochondria. In receptor-mediated mitophagy, the mitophagy receptors, such as BNIP3, BNIP3L, and FUNDC1, among others, localized in the outer mitochondrial membrane interact with MAP1LC3B on the growing mitophagosome. Unlike the PINK1-PRKN pathway, this mechanism is independent of ubiquitin. (B) alternative RAB9A-dependent mitophagy is induced under stress conditions in the heart. A complex of ULK1 and RAB9A initiates phagophore formation and recruits RIP1, which subsequently phosphorylates and activates DNM1L to mediate mitochondrial fission. This fission process segregates damaged mitochondria, promoting their selective removal via alternative mitophagy. Mitochondria are sequestered by RAB9A-associated membranes, which thereafter fuse with mitolysosomes for degradation and recycling. Created in BioRender. Nàger, M. (2025). https://BioRender.com/j9pw3qd.

Selective degradation of damaged or dysfunctional mitochondria by mitophagy utilizes some of the same cellular machinery as canonical autophagy. Mitophagy enables maintenance of a healthy pool of mitochondria, sustaining cellular ATP production and reducing oxidative damage from reactive oxygen species (ROS). Thus, a basal level of mitophagy is important in meeting and adapting to the metabolic needs of the cell^[48]^. Induction of mitophagy in response to stress may be beneficial and different cellular stress conditions elicit distinct mitophagy pathways, but the mechanistic insight is still limited^[49,50]^. Mitophagy pathways can be classified as either ubiquitin-dependent or independent, based on the selection mode for the mitochondria destined for degradation (Figure 3). The PINK1-PRKN-mediated mitophagy, driven by the enzyme 3 (E3) ubiquitin ligase PRKN (parkin RBR E3 ubiquitin protein ligase) and the protein kinase PINK1 (PTEN induced putative kinase 1) is the most extensively characterized mitophagy pathway^[51,52]^. When mitochondrial membrane potential is lost, PINK1, normally imported into mitochondria and degraded, accumulates in the outer membrane of mitochondria. PINK1 phosphorylation selectively recruits PRKN to damaged mitochondria, resulting in a feed-forward ubiquitylation of outer mitochondrial membrane proteins that recruits important mediators of mitophagy, including soluble SARs (Figure 3A)^[53]^. In contrast to PINK1-PRKN mediated mitophagy, receptor-mediated mitophagy is ubiquitin-independent and involves membrane bound SARs, present in mitochondrial membranes (Figure 3A). As for the soluble SARs, these receptors engage in LC3-interacting region (LIR) motif mediated interactions with the Atg8-family proteins on the forming mitophagosome. The most characterized mitophagy receptors are BNIP3 (BCL2 interacting protein 3), BNIP3L/Nix (BCL2 interacting protein 3 like) and FUNDC1 (FUN14 domain containing 1). Interestingly, BNIP3 and BNIP3L were initially assigned roles in promoting apoptosis^[54]^. BNIP3L mediated mitophagy is important in development of erythrocytes through coordinated clearance of mitochondria, also termed programmed mitochondrial degradation^[55,56]^. BNIP3 is linked to mitophagy under hypoxic conditions^[57]^. Notably, emerging evidence suggests that BNIP3 and BNIP3L also influence the basal level of mitophagy whereby their mitophagy activity is restricted in absence of stress^[58]^. BNIP3 and BNIP3L are expressed initially as inactive cytosolic monomers, but during stress conditions, such as hypoxia they can form stable homo- and hetero-dimers and insert into the outer mitochondrial membrane through their C-terminal domains^[59]^. In addition, LC3-interacing region (LIR) motif binding of both receptors to the Atg8-family proteins is positively regulated by phosphorylation, enhancing mitophagy^[60,61]^. FUNDC1, on the other hand, does not form dimers and its LIR binding to Atg8-family proteins is negatively regulated by phosphorylations that prevent mitophagy in normoxic conditions^[62]^. During hypoxia, PGAM5 (PGAM family member 5, mitochondrial serine/threonine protein phosphatase) dephosphorylates FUNDC1 and activates mitophagy^[62,63]^. Conversely, ULK1 mediated phosphorylation of FUNDC1 during hypoxia or mitochondrial depolarization can also enhance its LIR interaction to promote mitophagy^[64]^.

It is important to note that mitophagy can occur independently of canonical autophagy and altered levels of autophagy do not necessarily correlate with changes in mitophagy, and vice versa^[65–67]^. For instance, hearts of *fundc1* knockout (KO) mice display reduced mitophagy, while autophagy is largely unaltered^[68]^. Furthermore, autophagy in the mouse heart is upregulated earlier than mitophagy in mice fed with high-fat diet (HFD) as described below^[69]^.

An alternative mitophagy mechanism, mainly described in cardiomyocytes under metabolic stress, becomes active when conventional autophagy is inhibited, such as through deletion of *Atg7*. This pathway thus does not rely on Atg8-family protein lipidation^[13]^ (Figure 3B). Instead, alternative mitophagy involves the sequestration of damaged mitochondria in RAB9A (member RAS oncogene family)-associated autophagosomes via an ULK1-dependent mechanism, bypassing Atg8-family protein involvement. A large protein complex, consisting of ULK1, RAB9A, RIP1 (ralA binding protein 1) and DNM1L/DRP1 (dynamin 1 like) assembles at the mitochondria-associated endoplasmic reticulum membrane to facilitate the process^[70–72]^. DNM1L, which is critical for mitochondrial fission, is activated by phosphorylation at Ser616. Under stress conditions, ULK1 phosphorylates RAB9A at Ser179, initiating the formation of the ULK1-RAB9A-RIP1 complex, promoting DNM1L activation and thus mitochondrial fission^[73]^. However, the precise mechanism by which mitochondria are identified and tagged for degradation through alternative mitophagy remains unknown.

## Physiological importance of autophagy and mitophagy

Autophagy plays a crucial role in cardiovascular physiology, ensuring the stability and proper functioning of the heart and blood vessels. Studies in mice demonstrate that global deletion of essential autophagy-related genes, such as *Atg5, Atg7, Becn1*, *or Prkn*, results in embryonic and perinatal lethality, underscoring the importance of autophagy^[74–76]^. However, cardiac-specific knockouts (KO) of these genes in cardiovascular cells are not lethal at birth^[77]^. This distinction provides a unique opportunity for researchers to investigate the physiological role of autophagy and mitophagy in the cardiovascular system.

### Role of autophagy in cardiac development and during adult stage

The heart is the first organ to become functional during embryogenesis, and its formation is governed by intricate signaling pathways. The process starts with the differentiation of mesodermal cells into cardiac progenitor cells, which form the primitive heart tube. This tube undergoes looping, segmentation, and chamber formation to create the four-chambered structure of the heart^[78]^. Autophagy is required for normal heart development, playing a critical role in maintaining cellular integrity and supporting the dynamic processes orchestrating heart development. The initial observations of autophagosomes in fetal mouse cardiomyocytes using electron microscopy can be traced back to the 1970s^[79]^. In a zebrafish model expressing the GFP-LC3 reporter, basal levels of autophagy are observed in all the stages of embryonic cardiac development^[80]^. In addition, knockdown of autophagy-related genes, such as *atg5*, *atg7*, and *becn1*, in zebrafish models results in structural abnormalities in the heart and vascular system^[80]^.

Whole-body deletion of *Rb1cc1/Fip200* in mice also results in embryonic death, which is associated with severe liver dysfunction and heart failure. The cardiac phenotype is characterized by thinner ventricular walls and ventricular dilation^[81]^. Additionally, whole-body deletion of *Atg13* in mice results in embryonic lethality and myocardial defects, including thinning and disorganization of the ventricular wall. Interestingly, these effects appear to be independent of autophagy^[82]^. Perinatal cardiomyocyte-specific loss of either *Ulk1* or *Ulk2* in mice does not result in a decline in autophagy or cardiac function. However, loss of both results in impaired autophagy in the adult heart along with age-related cardiomyopathy and premature death. Conversely, inducible cardiac-specific KO mouse models of *Ulk1* or *Ulk2* indicate that adult loss of ULK1, but not of ULK2, results in lethal cardiac dysfunction with impaired autophagy and major cellular abnormalities. These results indicate that ULK1 undertakes a more prominent role in the adult heart^[83]^.

Autophagy is also crucial for preserving cardiac function and homeostasis after embryonic development. Autophagy dysregulation is implicated in various cardiac diseases during postnatal and adult life. In adult mice, temporally controlled cardiac-specific deletion of *Atg5* results in cardiac hypertrophy, left ventricular dilation, and contractile dysfunction. These pathological changes are accompanied by increased ubiquitination of proteins, disorganized sarcomere structures, mitochondrial misalignment, and formation of intracellular aggregates^[84,85]^. In contrast, mice with cardiomyocyte-specific overexpression of ATG7 exhibit elevated autophagy levels without causing any detectable cardiac abnormalities. These mice maintain normal cardiac anatomy and function throughout at least mid-adulthood^[86]^. Cardiac-specific overexpression of BECN1 also promotes autophagy, while haploinsufficiency of *Becn1* results in reduced autophagic activity and accumulation of damaged mitochondria. However, alterations in the level of BECN1 do not cause significant cardiac dysfunction under baseline conditions^[87]^. Notably, muscle-specific (skeletal muscle and heart) deletion of *Atg14* leads to hypertrophic cardiomyopathy, which is associated with abnormal accumulation of autophagic cargoes in the heart and early mortality^[88]^. Cardiac-specific deletion of *Atg3* also results in cardiac dysfunction and early mortality in mice, although it does not disrupt embryonic development. These mice exhibit impaired autophagic flux leading to defective mitochondrial function and reduction in the availability of the cellular metabolite nicotinamide adenine dinucleotide (NAD+)^[89]^.

Functional lysosomes are a prerequisite for autophagy flux. The protein LAMP2 (lysosomal-associated membrane protein 2) is one of the major components of the lysosomal membrane and is crucial for lysosome functions. Mutations in the *LAMP2* gene in humans are associated with Danon disease which presents symptoms such as hypertrophic or dilated cardiomyopathy, heart failure, muscle weakness, retinopathy, and cognitive impairment^[90]^. Notably, while *Lamp2*-deficient mice do not display significant cardiac abnormalities during early life, older mice exhibit excessive accumulation of autophagic vacuoles and impaired degradation of long-lived proteins. This leads to cardiomyopathy characterized by severe hypertrophy and contractile dysfunction^[91]^. In a more recent mouse model of Danon disease, with an in-frame exon 6 deletion mutation in the *Lamp2* gene found in a human family, an abnormal LAMP2 protein is expressed at a lower than usual level instead of being totally absent. These mice also have disrupted autophagy and develop hypertrophic cardiac remodeling, heart failure, and cardiac arrythmias^[92]^. Moreover, defects in autophagosome-lysosome fusion, mitochondrial abnormalities, and impaired contractility are also observed in hiPSC-derived cardiomyocytes from Danon disease patients^[93]^.

Iron is essential for oxygen transport and oxidative phosphorylation as well as other cellular processes. Imbalance in iron homeostasis, whether disrupted by iron excess or deficiency, has been implicated in the development of cardiomyopathy and heart failure. The TFRC (transferrin receptor) plays a crucial role in iron uptake via the transferrin cycle, facilitating receptor-mediated endocytosis of iron bound to serum transferrin^[94]^. Mice with cardiac-specific *Tfrc* deletion develop cardiac hypertrophy and die by post-natal day 11. These mice exhibit a marked reduction in mitochondrial electron transport chain complexes, along with abnormal mitochondria morphology, resulting in impaired mitochondrial biogenesis and mitochondrial insufficiency in the heart. Interestingly, their hearts show upregulation of proteins involved in the progression of mitophagy/autophagy pathways. For instance, ATG16L, a key protein involved in phagophore formation, is increased in *Tfrc*-deficient hearts. Additionally, expression of the ATG12-ATG5 complex and ATG4B, both critical for Atg8-family protein lipidation, is upregulated. However, the level of lipidated MAP1LC3B is decreased and a notable decrease in the expression level of mitophagy receptors, such as BNIP3L and FUNDC1, is found in hearts with reduced TFRC expression levels. These findings indicate that TFRC-deficient cardiomyocytes attempt to initiate mitophagy, however deficiency in the proteins involved in cargo recognition leads to ineffective mitophagy^[94]^.

MicroRNAs are important regulators of gene expression and impact on autophagy in the heart. Both microRNA (miR)-212 and microRNA (miR)-132 directly target the anti-hypertrophic and pro-autophagic FOXO3 (forkhead box O3) transcription factor^[95]^. Mice deficient of microRNA (miR)-212 and miR-132 have induced autophagy but normal heart function. However, cardiomyocyte overexpression of miR-212 and miR-132 in mice attenuates autophagy and leads to hypertrophy, heart failure, and death^[96]^. Likewise, cardiac-specific miR-199a overexpressing mice display impaired autophagy through indirect MTORC1 activation resulting in induced cardiac hypertrophy and heart failure^[97]^.

Collectively, these findings highlight the essential role of autophagy during cardiac development and in normal physiology.

### Role of mitophagy in cardiac development and during adult stage

Mitophagy is essential for cardiac development, as the deletion of key mitophagy components leads to significant defects in cardiac development^[98]^. Interestingly, germline deletion of *Prkn* in mice does not impair normal cardiac development and functional mitochondria are maintained during adulthood^[99]^. However, heart-specific deletion of *Prkn* during perinatal period (day1) in mice results in lethality^[100]^. In contrast, tamoxifen-mediated ablation of *Prkn* in the adult heart does not lead to cardiac and mitochondrial dysfunction^[101]^. These findings suggest that germline and adult-stage KO can activate compensatory mechanisms, whereas mitophagy is particularly critical during the perinatal period. The perinatal period is characterized by a metabolic transition from glycolysis to oxidative phosphorylation in mouse cardiomyocytes, highlighting the importance of mitophagy in supporting this metabolic shift^[98]^. Receptor-mediated mitophagy is also implicated in normal heart function. Overexpression of FUNDC1 increases mitophagy flux in mouse neonatal cardiomyocytes and gives rise to cardiac dysfunction. Conversely, deletion of *Fundc1* in cardiomyocytes results in impaired mitophagy, elongated mitochondria, compromised mitochondrial functions, and the development of cardiac dysfunction^[68,102]^. In a similar manner, deletion of both *Bnip3* and *Bnip3l* in mice leads to accelerated development of cardiac dysfunction linked to accumulation of dysfunctional mitochondria and impaired mitophagy^[103]^. Moreover, PRKN-independent mitophagy mediated by the mitochondrial ubiquitin ligase RNF7 (ring finger protein 7) is important for normal heart function. Ablation of *Rnf7* in adult mouse hearts results in severe mitochondrial dysfunction and heart failure linked to impaired mitophagy^[104,105]^. Mitochondrial quality control is maintained by continuous cycles of fusion and fission, collectively referred to as mitochondrial dynamics. Mitophagy is closely associated with mitochondrial fission, a process mediated by its key effector DNM1L, which also facilitates peripheral fission of damaged mitochondria, enabling their efficient engulfment by autophagosomes for degradation^[106]^. Loss of *Dnm1l* in cardiomyocytes leads to lethal cardiac dysfunction in mice. The *dnm1l* KO cardiomyocytes present enlarged mitochondria, impaired mitochondrial respiration, and disrupted mitophagy flux, preventing the clearance of dysfunctional mitochondria^[70,107]^. Mitochondrial fusion, on the other hand, is regulated by MFN1 (mitofusin 1) and MFN2 (mitofusin 2). Cardiac-specific deletion of both *Mfn1* and *Mfn2* in mice leads to fragmented mitochondria, decreased mitochondrial respiration and impaired myocardial contractile function^[108]^. Furthermore, expression of a phosphomutant MFN2 (MFN^T111A/S442A^) in mice inhibits PRKN translocation to the mitochondria and disrupts mitophagy. This leads to cardiac dysfunction and death by 7 or 8 weeks^[100]^. Finally, disruption of mitochondrial dynamics by a triple KO of *dnm1l*, *mfn1,* and *mfn2* in mice abolishes mitophagy, resulting in accumulation of morphologically and functionally abnormal mitochondria. This mitochondrial dysfunction drives cardiac hypertrophy with an increase in left ventricular mass, decreased cardiac function, and progression to heart failure^[109]^

### Role of autophagy in the vascular system

The vascular system is mainly composed of vascular smooth muscle cells (VSMCs) and vascular endothelial cells (VECs), which work together to maintain vascular function and integrity of the blood vessels. The vascular endothelium, composed of a monolayer of vascular endothelial cells (VECs) forms the inner lining of all vascular compartments. Vascular endothelial cells (VECs) are directly involved in detecting and reacting to signaling molecules in the blood and in sensing hemodynamics. Mechanical forces are an important modulator of endothelial autophagy. Shear stress, generated by the frictional forces of blood flow along the surface of the endothelium, triggers autophagy through mechanotransduction pathways in a reversible manner. This process is essential for the proper alignment of endothelial cells in the direction of blood flow, a well-known feature of healthy endothelium^[110,111]^. Nitric oxide (NO) is a key regulator of the vascular system in controlling vascular tone and blood circulation. Emerging evidence highlights a critical connection between autophagy and the regulation of nitric oxide (NO) bioavailability. Blocking autophagic flux, either through *Atg3* deletion or bafilomycin A_1_ treatment in VECs, disrupts endothelial NOS3/eNOS (nitric oxide synthase 3) phosphorylation and reduces NO production under shear stress^[112]^. Conversely, activating autophagic flux under steady laminar shear stress enhances eNOS expression, thereby supporting vascular homeostasis^[113]^. Additionally, shear stress activates SIRT1 (sirtuin 1), a nicotinamide adenine dinucleotide (NAD+)-dependent histone deacetylase, which promotes autophagy by deacetylating key autophagy-related gene promoters^[114]^.

TFEB plays a pivotal role in vascular development. Expression of constitutive knock-in TFEB-EGFP in mice shows that TFEB is expressed in early developing blood vessels and that its expression persists postnatally^[115]^. Deletion of *Tfeb* in VECs in mice results in embryonic lethality due to insufficient vascular development. Furthermore, inducible deletion of *Tfeb* in VECs after birth (d1 or d5) leads to a significant reduction in VEC proliferation and migration. Interestingly, expression of VEGFR (vascular endothelial growth factor receptor) is upregulated in *Tfeb*-deficient VECs. VEGFR is widely recognized as the master regulator of vascular development and angiogenesis in adult life^[116]^. Further analysis reveals that VEGFR trafficking is disrupted in *Tfeb*-deficient VECs, leading to its accumulation at the plasma membrane. This aberrant localization inhibits the VEGF (vascular endothelial growth factor) signaling pathway, thereby impairing vascular development in *Tfeb*-deficient mice^[115]^. Autocrine VEGFA (vascular endothelial growth factor A) plays a critical role in maintaining the integrity and survival of VECs. Depletion of cell-autonomous VEGFA signaling in VECs results in mitochondria fragmentation and a significant reduction in mitochondrial respiration capacity. VECs with *Vegfa* deletion exhibit an increase in autophagic vacuoles compatible with an increased autophagic flux. Interestingly, *Vegfa* deletion increases autophagic cell death, whereas silencing of *Atg7* rescues VECs from death. The cellular alterations caused by *Vegfa* deficiency result in pathological outcomes, including heart fibrosis and an increase in lesions within the cardiac vascular network^[117]^. In contrast, absence of pro-survival transcription factor KLF4 (KLF transcription factor 4) in VECs in mice suppresses autophagy. These KO mice display a diminished in vivo response to the vasodilator acetylcholine, indicating endothelial dysfunction^[118]^. Likewise, VEC-specific deletion of *Prkaa1* or *Prkaa2* in mice impairs autophagy, inhibits mitochondrial fragmentation and results in endothelial dysfunction. Notably, restoring autophagy through rapamycin treatment effectively reverses the endothelial dysfunction caused by *prkaa2* KO^[119]^. As part of their important function, VECs secrete different factors, and autophagy is also implicated here. One such factor is Von Willebrand factor (VWF), a glycoprotein essential for platelet adhesion at sites of vascular injury. VWF is frequently localized within autophagosomes in VECs. In human umbilical cord vascular endothelial cells (HUVECs), *Atg7* and *Atg5* silencing results in defects in the processing of the pro-VWF precursor to its active form, indicating that autophagy plays a pivotal role in the maturation and secretion of VWF. In mice, KO of *Atg7* does not affect the overall intracellular expression levels of VWF but significantly reduces the number of Weibel-Palade bodies, the intracellular organelles responsible for VWF secretion. Plasma analysis in *Atg7*-deficient mice reveals an increase in the non-active form of VWF. In addition, both pharmacological inhibition of autophagy or genetic KO of *Atg7* or *Atg5*, block the secretion of VWF, leading to prolonged bleeding times in mice^[120]^.

Vascular smooth muscle cells (VSMCs) form the medial layer of blood vessels and are specialized contractile cells that play a critical role in regulating vessel tone, blood flow, and the delivery of nutrients and oxygen to tissues^[121]^. Autophagy plays a pivotal role in the differentiation of epicardial progenitor cells into vascular smooth muscle cells (VSMCs). Treatment with rapamycin, for autophagy induction, significantly accelerates differentiation of epicardial progenitors into VSMCs. In contrast, inhibition of autophagy with 3-methyladenine delays this differentiation process^[122]^. VSMCs present high plasticity degree and can change their phenotype in response to local changes in the environment. Contractile VSMCs have spindle-shaped morphology and are characterized by the expression of contractile proteins. VSMCs can switch between phenotypes, allowing them to adapt to environmental signals, modulate vascular tone, drive growth, and contribute to repair mechanisms^[123]^. In contrast to contractile VSMCs, synthetic or secretory phenotypes are involved in vascular repair. Autophagy is essential for phenotypic switching in VSMCs, a process mediated in part by PDGF (platelet-derived growth factor). This growth factor not only induces phenotypic switching but also activates autophagy. When autophagy is inhibited with 3-methyladenine prior to PDGF treatment, phenotypic switching is completely abolished, demonstrating that PDGF-induced autophagy is a prerequisite for this cellular phenotype transformation^[124]^. Disruption of essential autophagy-related genes further emphasizes the critical role of autophagy in maintaining VSMCs function, and overall vascular health. For instance, *Atg7* deletion in VSMCs impairs autophagy, resulting in reduced proliferation capacity and the onset of cellular senescence^[125,126]^. Additionally, these cells present disrupted Ca^2+^ homeostasis, leading to imbalances in Ca^2+^ signaling, and impaired vascular contraction in VSMCs^[125]^. Moreover, deletion of *Atg7* in VSMCs in young mice leads to an increase in arterial stiffness and atherosclerosis, a key indicator of vasculature dysfunction^[127]^.

Together, these findings illustrate the intricate role of autophagy in vascular health, from regulating cellular differentiation and phenotypic modulation to maintaining vascular structure and function.

## Implications of autophagy and mitophagy in cardiovascular aging

Aging disrupts cardiovascular homeostasis by driving gradual structural remodeling and functional alterations in the heart and the vasculature, thereby increasing susceptibility to various cardiovascular diseases. In otherwise healthy individuals, aging in the heart is characterized by left ventricular wall thickening and myocardial fibrosis^[128]^. These structural changes contribute to cardiac hypertrophy and increase myocardial stiffening, which, although initially adaptive, ultimately results in a decline in diastolic function, one of the hallmarks of cardiac aging^[129]^.

### Role of autophagy in cardiovascular aging

A decline in the expression level of autophagy markers is described in the vasculature of aged mice and humans^[130]^ and diminished autophagy is regarded as a key feature of aging within the cardiovascular system^[131]^. While aging affects all cell types in the cardiovascular system, cardiomyocytes are particularly vulnerable due to their terminally differentiated state and lack of proliferative capacity. Cardiomyocytes must sustain continuous heart contractions throughout an individual’s lifespan, rendering them susceptible to age-related dysfunction and failure^[132]^.

However, the role of autophagy in the aging heart remains a topic of debate, with early studies reporting contradictory results. An inhibition of autophagy in the aging heart is evidenced by decreased MAP1LC3B-II levels in wild-type C57Bl/6J mice aged 26 months compared to 10 weeks^[133]^. Conversely, MAP1LC3B-II levels remain constant when comparing 3 to 20–24-month-old FVB mice^[134]^. Furthermore, an increase in MAP1LC3B-II and BECN1 levels is detected in C57BL/6 mice aged 18 months compared to 2 months^[135]^. Notably, these studies primarily rely on measurements of autophagosome abundance, based on MAP1LC3B-II measurements. Recent studies using autophagy inhibitors suggest that autophagic flux is impaired in cardiac tissue during aging^[136–138]^. Despite these advances, a comprehensive investigation specifically evaluating autophagy flux in the aging heart is still pending.

One identified key factor in age-related decline of autophagy is the reduced activity of sirtuin deacetylases, which leads to increased acetylation of ATG proteins, such as ATG5, ATG7, and Atg8-family proteins. This heightened acetylation consequently diminishes their activity^[139–141]^. Mice with cardiac-specific *Atg5* deletion are born without apparent abnormalities, but they die prematurely. Cardiac *Atg5*-ablation in mice results in structural and functional mitochondrial defects accompanied by impaired excitation-contraction coupling. These pathological changes lead to progressive chamber dilation and cardiac dysfunction as the mice age. Consequently, these mice develop age-related cardiomyopathy^[133,142]^. Notably, whole-body overexpression of ATG5 in mice extends median lifespan. These animals also exhibit higher levels of autophagy in the heart. Moreover, near the end of their lifespan, the hearts of ATG5 overexpressing mice display reduced age-related changes and cardiac fibrosis compared to age-matched WT (wild type) mice controls, indicating that ATG5 overexpression protects against age-associated cardiac remodeling^[143]^. Similarly, mice harboring a mutation in *Becn1* (F121A) that reduces the interaction between BCL2 (BCL2 apoptosis regulator) and BECN1 exhibit increased basal level of autophagy and prolonged lifespan^[144]^. In addition, old mice with this BECN1 mutation display increased autophagic flux mitigating age-related phenotypes^[145]^.

The PtdIns3K-C2 member UVRAG regulates autophagy and endocytosis. Deletion *of Uvrag* leads to the accumulation of autophagosomes and impaired autophagy flux. In *Uvrag*-deficient mice, there is an increased number of lysosomes, as evidenced by elevated levels of LAMP1 (lysosomal associated membrane protein 1) and LAMP2 (lysosomal associated membrane protein 2) and an increase in lysosome-positive structures in the hearts. These findings indicate defective autophagic flux, likely due to suppression of autophagosome-lysosome fusion. Interestingly, no significant differences are observed between WT and *uvrag* KO mice at an early age. However, by 10 months of age, *Uvrag*-deficient hearts exhibit pathological changes, including hypertrophy, interstitial cardiac fibrosis, and increased apoptosis, suggesting that these mice develop age-related cardiomyopathy^[146]^. FBXO32/ATROGIN 1 (F-box protein 32) is a muscle-specific ubiquitin ligase and a key regulator in the ubiquitin-proteasome system under the control of FoxO (Forkhead box protein O) transcription factors^[147]^. FBXO32 is closely associated with hypertrophy and progression of heart disease. Interestingly, mice with cardiomyocyte-specific KO of *fbxo32* develop an age-related cardiomyopathy with interstitial fibrosis. The fibrotic areas in *Fbxo32*-deficient hearts display elevated endoplasmic reticulum (ER) stress, which promotes cardiomyocyte apoptosis. Notably, *Fbxo32*-deficient cardiomyocytes show an accumulation in the abundance of autophagosomes and lysosomes, indicating impaired autophagic flux^[147]^. Moreover, *Fbxo32* deletion reduces degradation of CHMP2B (charged multivesicular body protein 2B), an ESCRT-III protein essential for lysosome biogenesis and autophagosome-lysosome fusion. Disrupted CHMP2B turnover induces CHMP2B aggregates, hinders endosome maturation, and prevents autophagosome-lysosome fusion, leading to impaired autophagic flux^[147]^.

Deletion of core autophagy genes also leads to dysfunction in VSMCs during aging. Specifically, *Atg7* deficiency causes SQSTM1 accumulation, promoting cellular senescence and contributing to development of atherosclerosis. Interestingly, overexpression of SQSTM1 induces a similar phenotype as *Atg7* deletion, suggesting that SQSTM1 accumulation is a key driver of VSMC senescence^[148]^. Although ECs exhibit higher proliferative capacity and reduced predisposition to accumulate damage during aging, they remain highly reliant on autophagy for the clearance of misfolded proteins and damaged organelles. KLF4 protein levels decrease with age in the human endothelium. Notably, overexpression of KLF4 in human umbilical cord vascular endothelial cells (HUVECs) induces autophagic flux. Furthermore, endothelium-specific overexpression of KLF4 in mice reduces aging-associated increase in vessel stiffness, an effect that is reversed upon treatment with the autophagy inhibitor chloroquine. Taken together, these data indicate a link between KLF4 regulated autophagy, endothelial dysfunction, and blood vessel aging^[118]^.

### Role of mitophagy in cardiovascular aging

The accumulation of damaged and dysfunctional mitochondria is a hallmark of the aging heart and is strongly associated with age-related cardiovascular decline^[131]^. Mechanistically, accumulation of dysfunctional mitochondria is thought to result from age-related decline of mitophagy^[149]^. In support of that, deletion of *Pink1* in mice leads to an age-dependent accumulation of damaged mitochondria, resulting in premature development of cardiac hypertrophy and systolic dysfunction due to impaired mitophagy^[150,151]^. Moreover, deletion of *Tp53* (tumor protein p53) suppresses cardiac aging in mice linked to enhanced mitophagy due to alleviated TP53 inhibition of PRKN^[152]^. Similarly, impaired endothelial mitophagy, observed in *prkaa2* KO mice, is associated with mitochondrial fragmentation and vascular endothelial dysfunction. These defects can be alleviated by ATG7 overexpression or by administering rapamycin^[119]^. However, the mechanisms underlying the decline in mitophagy with age remain poorly understood, likely due to the involvement of multiple pathways and complex regulatory networks.

## Autophagy and mitophagy in cardiovascular disease

In response to stress, autophagy and/or mitophagy can be induced or suppressed. Although an adaptive induction of these processes is generally considered protective, both excessive and impaired degradation of cellular constituents through these pathways is associated with pathological processes in the development of cardiovascular disease. Reported involvement of autophagy or mitophagy in vascular disease (atherosclerosis), myocardial infarction (MI) and ischemia-reperfusion injury, heart failure as well as doxorubicin-induced cardiotoxicity will be described in the following sections.

## Atherosclerosis

In atherosclerosis, a chronic inflammatory vascular disease, the arterial wall gets thicker and stiffer due to the formation of a fibrous lipid/cholesterol-enriched plaque. The vascular endothelium constituting the arterial lumen surface is constantly subjected to shear stress derived from blood flow. Plaque formation is induced by lipid deposition and accumulation within the arterial subendothelial space, resulting in an inflammatory response. Heart blood perfusion is hampered as atherosclerosis progresses due to narrowing of the arterial lumen. This also predisposes to plaque rupture or erosion and thromboembolism which can lead to myocardial infarction or stroke. Atherosclerosis is in fact the pathological basis of most cardiovascular diseases^[153,154]^. Impairment of the protective endothelial function drives atherogenesis, encompassing increased permeability, formation of focal lesions, and a pro-inflammatory state^[155]^. Within the vasculature, predisposed atherogenic sites are where perturbation of the laminar shear stress occurs, e.g. at sites of bifurcations and inner curvatures^[156]^. Traditional cardiovascular risk factors such as dyslipidemia, hypertension, obesity, and arterial wall shear stress contribute to endothelial dysfunction and damage by facilitating accumulation of circulating plasma lipoproteins (low-density lipoproteins, LDL in particular) into the subendothelial space^[157,158]^. In the artery wall, low-density lipoprotein becomes oxidized (OxLDL) and aggravates endothelial cell dysfunction by recruitment of circulating monocytes and migration of tissue-resident macrophages and vascular smooth-muscle cells (VSMCs)^[159]^. Through their scavenger receptors, these cells engulf oxidized LDL resulting in lipid-laden foam cells, a major characteristic of atherosclerosis^[160,161]^. Cell death within the plaque increases with progression of atherosclerosis and finally outpaces efferocytosis (removal of apoptotic cells), leading to an accumulation of apoptotic foam cells in the necrotic core of the plaque^[162]^. Such advanced lesions get covered by a fibrous cap consisting of proliferative VSMCs secreting fibrous and calcified extracellular matrix constituents^[163]^. This is accompanied by infiltration of immune cells (circulating monocytes and migration of resident macrophages), further contributing to vascular inflammation in advanced plaques^[164]^. The predominant animal models of atherosclerosis since the 1990s are the LDLR (low-density lipoprotein receptor)-deficient mice (*ldlr*^−/−^) and the *apoe* (apolipoprotein E) KO (*apoe*^−/−^) mice. Both strains have elevated levels of plasma lipoproteins and form atherosclerotic plaques, and high-fat diet (HFD) is often used to promote plaque formation^[165]^.

### Role of autophagy in vascular cells in the development of atherosclerosis

The endothelial monolayer functions as a semi-permeable barrier as well as regulator of vessel homeostasis. Autophagy in VECs contributes to the adaptive response to laminar flow and shear stress of blood. Laminar shear stress promotes efficient autophagy in VECs, limiting oxidative stress, inducing NOS expression, and inhibiting inflammation^[113,114,166]^ However, in atherogenic sites with low or perturbed shear stress, the endothelial cells display defective autophagy. This contributes to inflammation, senescence, and cell death and thus progression of atherosclerosis. In hypercholesterolemic mice with an atheroprone background (*apoe*^−/−^), endothelial cell-specific deletion of *Atg5* or *Atg7* aggravates the atherosclerotic burden and increases inflammation^[110,120]^. Conversely, increased endothelial autophagy, as a result of *Cav1* (caveolin 1) deficiency in *ldlr*^−/−^ mice fed with HFD is athero-protective^[167]^. Furthermore, activation of endothelial autophagy with rapamycin in *apoe*^*−/−*^ mice leads to exosome-mediated delivery of microRNA miR-204-5p to VSMCs, attenuating HFD-induced endothelial cell apoptosis and VSMCs calcification^[168]^.

VSMCs constitute the main component of the vessel wall and proper cellular function is essential for protecting the vessel wall against proatherogenic stimuli. Following vascular injury and inflammation, VSMCs can adopt a wide range of phenotypes and alternate between those in response to environmental cues. Such phenotypic switching usually refers to de-differentiation of quiescent contractile VSMCs to a proliferative and migratory synthetic state but can also apply to a phenotypic change to an alternative non-contractile phenotype^[169]^. VSMCs are found both in the fibrous cap and plaque core and their de-differentiation is a critical step in atherosclerotic plaque development. Upon cholesterol loading, VSMCs can transform into foam cells^[170]^. Interestingly, autophagy is involved in phenotypic switching of VSMCs. Activation of the P2RY12 (purinergic receptor P2Y12) receptor in atheroprone (*apoe*^−/−^) mice on HFD inhibits autophagy in VSMCs, reducing cholesterol efflux and promoting foam cell transformation^[171]^. Genetic disruption of essential autophagy proteins in VSMCs is also atherogenic. Specific deletion of *Atg7* in VSMCs leads to accelerated senescence, neointima formation, and diet-induced atherogenesis in atheroprone (*apoe*^−/−^) mice^[148]^. Defective autophagy in VSMCs through *Atg7* deletion in the same mouse model also enhances cell death, atherosclerotic plaque progression, and arterial outward remodeling, associated with increased frequency of aortic aneurysm rupture^[126]^. Of note, mice with VSMC-restricted *Atg5* deletion display increased incidence and severity of aortic dissection^[172]^. Plaque instability and the risk of plaque rupture are also increased in the context of *Atg7* knock out in VSMCs in atheroprone (*apoe*^−/−^) mouse model of plaque instability, indicating involvement of defective autophagy^[173]^.

Macrophages transform into foam cells through engulfing OxLDL or other modified lipoproteins. Here, increased uptake and reduced efflux of cholesterol mediate abnormally high cellular levels of cholesteryl esters stored within cytoplasmic lipid droplets^[174]^. Lipophagy is a form of selective autophagy that mobilizes cholesteryl esters and triglyceride from lipid droplets to lysosomes for degradation^[175,176]^. Hence, lipophagy is involved in cholesterol homeostasis and plays an important role in the pathogenesis of atherosclerosis. Interestingly, cholesterol gets sequestered within lysosomes in foam cells of advanced plaques, rendering it unavailable for extracellular metabolism. It is unclear whether this is positive or negative in terms of atherosclerosis, but the transition from a more benign to more advanced and complicated plaque coincides both temporally and spatially with lysosomal lipid accumulation in macrophages. This suggests that the lysosomal lipid accumulation in foam cells is a critical mediator of plaque development and may contribute to the transition toward more unstable plaque phenotypes^[177]^. Such lysosomal lipid accumulation impairs lysosome function and thus impacts on lipophagy, contributing to foam cell lipid overload^[178]^. Deletion *of Atg5* in macrophages hampers lipid droplet degradation through impaired lysosomal lipid hydrolysis^[179]^. Furthermore, macrophage ATG5 deficiency promotes plaque development and disease progression in mice on HFD^[180,181]^. Further evidence on the regulatory role of autophagy in atherosclerosis in mice is shown with macrophage-specific TFEB overexpression. TFEB can increase lysosomal lipid catabolism, lipolysis, and cellular fatty-acid oxidation^[182]^. In atheroprone (*apoe*^−/−^) mice, overexpression of TFEB results in decreased plaque formation, while the effect is abolished in mice with impaired macrophage autophagy through ATG5 or SQSTM1/p62 deficiency^[183]^.

### Role of mitophagy in vascular cells during development of atherosclerosis

Mitophagy is implicated in response to stress in obese and diabetic mice due to an increased expression of PINK1 and PRKN in all layers of the aortic wall, including the endothelium. Notably, no direct measurement of mitophagy is conducted in this study^[184]^. An increase in proliferation of VSMCs and exacerbated atherosclerotic lesions are also correlated with increased PINK1-PRKN mediated mitophagy in *apoe*^−/−^ mice. Thus, excessive activation of mitophagy can be detrimental in the development of atherosclerosis^[185]^.

High-protein diets also favor increased atherogenesis^[186,187]^. Raised levels of amino acids in blood and tissue stimulate MTORC1 activation in plaque macrophages. Furthermore, deletion of *Rptor*, a critical MTORC1 component, leads to reduced development of high-protein intake induced atherogenic plaque in atheroprone (*apoe*^−/−^) mice. The protective effect is attributed to restored degradation of dysfunctional mitochondria by mitophagy. Notably, the effect of *Rptor* deletion is entirely abrogated in mice with impaired macrophage autophagy through *atg5* KO^[188]^. In mice with a deletion of *Naxe/Aibp* (NAD(P)HX epimerase) and subjected to *Ldlr* knockdown and HFD, macrophages in atherosclerotic lesions display downregulated autophagy and increased apoptosis. Disrupted PRKN-dependent mitophagy is implicated since NAXE/AIBP interacts with PRKN and *in vitro* exposure of isolated macrophages from *aibp*^−/−^mice with OxLDL attenuates mitophagy^[189]^.

## Myocardial infarction and ischemia-reperfusion injury

Myocardial ischemia is caused by an inadequate blood flow to the myocardium. Cell death in the heart due to prolonged ischemia leads to myocardial infarction (MI) and the size of the infarct correlates to the duration of ischemia^[190–192]^. Reperfusion refers to restoration of blood flow after ischemia and timely reperfusion is the best therapeutic option for patients with myocardial infarction (MI)^[193]^. Paradoxically, reperfusion can also introduce additional injury, termed ischemia/reperfusion injury encompassing cellular processes such as Ca^2+^ and proton overload, oxidative stress, and mitochondrial dysfunction^[194]^. The most widely used method to model MI in animals involves surgical ligation of the left anterior descending coronary artery (LAD). For ischemia and reperfusion (I/R) modeling, the LAD is temporarily occluded before removing the suture to restore blood flow and mediate reperfusion of the affected tissue^[195]^.

### Role of autophagy in myocardial ischemia and reperfusion

The level and alterations in autophagy during myocardial ischemia and reperfusion have been investigated in both small and large animal models. In general, induction of autophagy in ischemia is regarded as beneficial, counteracting nutrient and oxygen deprivation. In a pig model of chronic ischemia, autophagy in the pig heart is induced and the level of apoptosis is diminished^[195]^. Prolonged ischemia (through LAD restriction for 3 hours) in a mouse model indicates that RHEB is inactivated. RHEB activates MTORC1 and thus inhibits autophagy. Cardiac-specific overexpression of RHEB leads to a significant increase in MTORC1 activity, both at baseline and during prolonged ischemia. In addition, the infarct size increases significantly^[196]^. Similarly, in mice with HFD–induced obesity and metabolic syndrome (HFD mice), MTORC1 activity is greater at baseline compared to control mice and it remains high during prolonged myocardial ischemia. Accordingly, autophagic activity in the heart is significantly suppressed in HFD mice, contributing to an increased ischemic injury. Notably, a reduction in susceptibility to ischemic myocardial damage can be achieved through pharmacological and genetic inhibition of MTORC1^[196]^. In agreement with this, MTORC1 activation through GSK3B (glycogen synthase kinase 3 beta) inhibition prevents autophagy in a detrimental manner during prolonged ischemia (2 hours) in mice. Also, impairment of autophagy through cardiac-selective deletion of *Nox4* (NADPH oxidase 4) aggravates ischemic injury during prolonged ischemia in the mouse heart^[197]^. Interestingly, after 2 hours of ischemia in mice, autophagy flux is partially impaired, displayed by a diminished increase in autophagosome abundance in the presence of chloroquine compared to sham operated mice^[198]^. Sustained ischemia in mice with permanent LAD ligation also impairs autophagy flux in cardiomyocytes, resulting in exacerbated post-infarct adverse cardiac remodeling. The remodeling effect can be attenuated through treatment with rapamycin (an autophagy enhancer) or further exacerbated with 3-methyladenine (an autophagy inhibitor)^[197]^. Of note, treatment with bafilomycin A_1_ in a similar mouse MI model also significantly increases infarct size^[199]^. Furthermore, *Adipoq* (adiponectin, C1Q and collagen domain containing) deficient mice display impaired autophagic flux and exacerbated ischemia-induced cardiac dysfunction^[200]^.

In contrast to ischemia, autophagy activation during reperfusion can be harmful or protective. In a mouse model with 20 min of ischemia and 20 min of reperfusion, autophagy is induced in ischemia and amplified even further in reperfusion^[201]^. Notably, during ischemia autophagy induction is PRKAA dependent, while during reperfusion, PRKAA is not active. Interestingly, BECN1 is upregulated in a ROS-dependent manner during reperfusion and both autophagy and myocardial injury are attenuated in mice with a heterozygous KO of *Becn1*^[201]^. This indicates that BECN1 is an important mediator of autophagy during reperfusion and that autophagy induction during reperfusion may be detrimental. In support of this, inhibition of autophagy during I/R, through microRNA miR-188-3p mediated impairment of ATG7 translation, results in a significant reduction in myocardial infarction size^[202]^. Similarly, inhibition of GSK3B protects against reperfusion injury through MTOR-dependent downregulation of autophagy^[203]^. In a mouse model of ischemia for 30 min followed by reperfusion for 90 min, autophagy flux during reperfusion is inhibited, demonstrated by a lack of a further increase in autophagosome abundance in the presence of chloroquine^[198]^. This indicates that hampered clearance of autophagosomes during reperfusion contributes to cell death. Hence, induction of autophagy during reperfusion could confer myocardial protection. Indeed, suppression of MST1 (macrophage stimulating 1), a serine-threonine kinase, during myocardial infarction is cardioprotective. MST1 inhibits autophagy through phosphorylation of BECN1, attenuating PtdIns3K-C1 formation. At the same time, MST1 phosphorylation induces the interaction of BECN1 with BCL2 and thus promotes apoptosis^[204]^. Furthermore, mice with conditional cardiomyocyte deletion of *Atg7* display impaired autophagy and aggravated I/R injury^[205]^. Taken together, regulation of autophagy during myocardial reperfusion is multilayered, and the cardioprotective effects of inhibition or induction of autophagy are likely context dependent.

Excessive activation of autophagy can result in cell death in a process termed autosis^[206]^. Myocardial I/R can induce autosis in cardiomyocytes. In a mouse model of 30 min of ischemia followed by 2–24 hours of reperfusion, autophagic flux is increased during early reperfusion but is inhibited during late reperfusion. The level of RUBCN (rubicon autophagy regulator), a negative regulator of autophagy, is significantly increased 6 hours after reperfusion, correlating with the timepoint when autophagy flux is impaired. RUBCN inhibits fusion of autophagosomes with lysosomes^[207,208]^ and downregulation of RUBCN improves autophagic flux during the late phase of reperfusion. Here, accumulation of autophagosomes is reduced and autosis and myocardial injury are diminished^[209]^. Interestingly, TFEB is activated in cardiomyocytes and can promote autosis during the late phase of reperfusion and thus contribute to reperfusion injury^[210]^.

### Role of mitophagy in myocardial ischemia and reperfusion

In the constantly beating and energy-demanding cardiomyocytes, mitochondria serve a crucial function. Mitochondria swelling, reduced number of cristae, and mitochondrial matrix clearing are ultrastructural morphological changes of damaged mitochondria that are widely accepted as cellular hallmarks of I/R injury^[211,212]^. Removal of such impaired mitochondria by mitophagy is crucial to prevent or limit cell death activation. Damaged mitochondria can undergo DNM1L/DRP1 mediated fission, membrane depolarization, accumulation of PINK1, and recruitment of the E3 ubiquitin ligase PRKN, promoting ubiquitination of mitochondrial proteins and finally elimination through PINK1-PRKN dependent mitophagy^[53]^. Autophagosomes containing mitochondria are absent in mice with heterozygous conditional KO of *dnml* after I/R and the infarct size is increased, indicating an important role of DNML1 in mediating removal of damaged mitochondria^[107]^. Ex vivo hearts from *pink1* KO mice subjected to 35 minutes regional ischemia followed by 30 minutes reperfusion show larger infarct size compared to control mice^[151]^. It should be noted that in these studies, direct assessment of mitophagy is not conducted. However, *prkn* KO mice subjected to MI by permanent ligation of the LAD display impaired mitophagy and exacerbated size of myocardial infarction^[99]^. Collectively, these results point toward the importance of functional PINK1-PRKN mitophagy during MI. In addition, other mitophagy mechanisms are activated in response to I/R. During ischemia in mice, an alternative mitophagy pathway dependent upon ULK1 and RAB9A instead of ATG7 is upregulated and protects the heart. In knock-in mice expressing phosphorylation-resistant RAB9A, ischemia-induced mitophagy activation is selectively attenuated resulting in an increase in infarct size. Notably, induction of macroautophagy is unaffected, illustrating that mitophagy and autophagy can be mediated by distinct mechanisms in vivo^[73]^. Receptor-mediated mitophagy is also important during myocardial I/R. In response to ischemia in mice, the level of inactive phosphorylated FUNDC1 (p-FUNDC1^Tyr18^) is dramatically downregulated, indicative of induction of FUNDC1-dependent mitophagy^[213]^. Conversely, FUNDC1-mediated mitophagy is suppressed during reperfusion through CK2α (casein kinase 2 alpha) dependent phosphorylation and inactivation of FUNDC1 (p-FUNDC1^Tyr13^), leading to progression of I/R injury^[213]^. Notably, systemic KO of FUNDC1 aggravates myocardial infarction while FUNDC1 overexpression induces mitophagy and reduces infarct size^[68]^. Further evidence for suppression of mitophagy during reperfusion comes from a study on the zinc transporter SLC39A7/ZIP7 (solute carrier family 39 member 7) which is upregulated at reperfusion. Upregulation of SLC39A7/ZIP7 reduces mitochondrial Zn^2+^ resulting in hyperpolarization. This suppresses mitophagy through preventing PINK1 and PRKN accumulation on mitochondria. KO of *Slc39a7/Zip7* enhances mitophagy at reperfusion and reduces ROS generation as well as infarct size in mouse hearts^[214]^. Notably, PGAM5 is also linked to mitophagy in the heart^[215]^. PGAM5 is involved in both PINK1-PRKN mediated mitophagy and receptor-mediated mitophagy^[216]^. A cardiac-specific KO of *Pgam5* results in induced cell death by necroptosis after ischemia and reperfusion, indicating a protective role of PGAM5, although direct mitophagy assessment in the heart is not demonstrated^[215]^. The involvement of PGAM5 in cardiac mitophagy is, however, unresolved as a recent study shows that its upregulation facilitates necroptosis during myocardial I/R injury in mice, whereas it's depletion does not rescue attenuated mitophagy upon I/R injury^[217]^. In contrast to the above-mentioned mouse studies where mitophagy appears to be suppressed during reperfusion, we recently demonstrated that mitophagy is significantly induced in human engineered heart tissue after I/R simulation in a ULK1-dependent manner^[218]^. More research is warranted to investigate the mitophagic response to I/R and map the underlying molecular mechanisms.

## Heart failure

Heart failure (HF) is a clinical syndrome due to a structural and/or functional impairment of ventricular filling or ejection of blood. Heart failure (HF) is subdivided into groups based on the measurement of left ventricular ejection fraction (LVEF). Heart failure (HF) with reduced ejection fraction (HFrEF) where LVEF is defined as ≤40% and HF with preserved ejection fraction (HFpEF) where LVEF ≥50% are most common^[219]^. HFpEF is often associated with metabolic disorders such as obesity, diabetes, and hypertension and accounts for over 50% of all HF cases^[220]^.

Pressure overload (PO) is a major contributor to HFrEF progression. Thus, for studying HFrEF development, a commonly used preclinical mouse model is a pressure overload (PO)-induced HFrEF by transverse aortic constriction (TAC)^[221,222]^. HFrEF also develops by 6 weeks after infarction by permanent LAD ligation in mice and I/R injury can result in HFrEF^[221]^. Hypertension, causing myocardial stiffening and diastolic dysfunction, is one of the main underlying conditions leading to HFpEF^[221]^. Administration of angiotensin II in mice is commonly used for studying hypertension-induced HFpEF. In addition, feeding with HFD or use of diabetic mouse models is also exploited^[221]^. Recently, two-hit mouse models with a combination HFD and constitutive NOS inhibition by N^[w]^-nitro-l-arginine methyl ester (L-NAME)^[223]^ or HFD and virus overexpression of renin^[224]^ are being used to study HFpEF. The role of autophagy and mitophagy is mostly described in HFrEF animal models, while the importance of these processes in HFpEF remains largely elusive.

### Role of autophagy in heart failure

Autophagy seems to play a dual role as detrimental or beneficial in pressure overload (PO)-induced HFrEF. Heterozygous disruption of *Becn1* results in diminished pathological remodeling induced by PO, while BECN1 overexpression accentuates cardiac dysfunction^[225]^. This indicates that inhibition of autophagy can have a protective effect against PO. On the other hand, cardiac-specific conditional inactivation of *Atg5* in a mouse model of TAC results in severe contractile dysfunction and left ventricle dilation 1 week after TAC and death due to HF thereafter^[84]^. Likewise, heterozygous deletion of *Atg5* in mice aggravates angiotensin II-induced cardiac hypertrophy^[226]^. In a mouse model of HFrEF induced by PO, activation of autophagy through a PRKG1 (protein kinase cGMP-dependent 1)-TSC2-RHEB complex mediated inhibition of MTORC1 is cardioprotective^[227]^. Homozygous deletion of *Fyco1* (FYVE and coiled-coil domain autophagy adaptor 1) results in impaired cardiac function during PO due to attenuated induction of autophagy^[228]^. FYCO1 is involved in microtubule plus end-directed transport of autophagic vesicles^[229]^. Interestingly, FYCO1 overexpression rescues cardiac dysfunction in mice in response to biomechanical stress^[228]^. Furthermore, induction of autophagy by rapamycin can suppress HFrEF progression in rats^[230]^. Mice with a deletion of *Dnase2*, (deoxyribonuclease 2, lysosomal), a lysosomal DNAse responsible for autophagic degradation of released mitochondrial DNA, display severe cardiac dysfunction associated with premature death after TAC induced PO, pointing towards the importance of functional autophagy^[231]^. Conversely, *Ficd* (FIC domain protein adenylyltransferase) deletion in mice prevents cardiac hypertrophy, fibrosis, and HF following TAC. FICD catalyzes addition of AMP (AMPylation) on its target proteins. At the cellular level, *ficd* KO enhances autophagic flux in cardiomyocytes subjected to TAC and endoplasmic reticulum (ER) stress. Selective degradation of the endoplasmic reticulum (ER) through ER-phagy is essential for preserving ER homeostasis and facilitating recovery following ER stress^[232]^. Interestingly, absence of FICD prevents AMPylation of HSPA5 (Heat shock protein family A(Hsp70) member 5 also known as BiP), a key regulator of the unfolded protein response (UPR) activated in response to ER stress. Unmodified HSPA5 interacts with RETREG1 (reticulophagy regulator 1), an ER-phagy receptor, thereby promoting ER-phagy. This highlights the role of ER-phagy in mitigating ER stress in HF^[233]^.

MicroRNAs also play a role in HF. Expression of miR-212 and miR-132 in the heart is induced by hypertrophic stimuli. Mice deficient of miR-212 and miR-132 display induced autophagy and are protected from PO-induced HF^[96]^. Conversely, in a mouse model of angiotensin II-induced cardiac hypertrophy, miR-93 levels are reduced resulting in inhibition of autophagy. SYT7 (synaptotagmin 7) is a high-affinity calcium sensor and a direct target of miR-93. Reduced levels of miR-93 in response to angiotensin II treatment result in an increase of SYT7 and consequent inhibition of autophagy. Overexpression of miR-93 or SYT7 inhibition rescues autophagy and preserves cardiac function^[234]^.

Genetic aberrations can cause cardiomyopathies such as desmin-related cardiomyopathy where an R120G missense mutation in the desmin chaperone CRYAB (crystallin alpha B; CRYAB^R120G^) is the best studied. Here, intracellular accumulations of protein aggregates are detected in cardiomyocytes^[235,236]^. Cardiac-restricted transgenic expression of CRYAB^R120G^ is sufficient to recapitulate dilated cardiomyopathy and cause heart failure in a mouse model^[237]^. Interestingly, induced cardiomyopathy progression and decreased cardiac function result from hemizygous KO of *Becn1* in CRYAB^R120G^ mice^[238]^. In contrast, autophagy activation by ATG7 overexpression and/or exercise in CRYAB^R120G^ mice attenuates the morphological and functional pathologies^[86]^. Alterations in autophagy is described in a wide range of cardiomyopathies, and for a comprehensive overview we direct the reader to an excellent review by Zech et al.^[239]^.

### Role of mitophagy in heart failure

Mitochondrial dysfunction with increased oxidative stress and reduced rate of ATP synthesis is a cellular hallmark of cardiomyocytes in end-stage HF patients^[240]^. Thus, degradation of compromised mitochondria through mitophagy is crucial. Analysis of the expression of PRKAA/AMPK isoforms in tissue biopsies from HF patients and healthy donors indicates an isoform switch from PRKAA2 to PRKAA1 during the progression of HF. PRKAA2 enhances mitophagy through PINK1 phosphorylation and overexpression of PRKAA2 prevents the development of TAC-induced HFrEF in mouse hearts. Conversely, *prkaa2*^−/−^ KO mice exhibit a decrease in mitophagy along with an exacerbation of cardiac dysfunction^[241]^. Thus, PINK1-dependent mitophagy appears to be blunted in response to PO. Interestingly, autophagy and mitophagy are only temporarily activated upon PO in mice and decline significantly thereafter, promoting development of HFrEF. Injection of the autophagy inducing peptide Tat-BECN1^[242]^ reactivating autophagy, attenuates HF induced by PO. Furthermore, haploinsufficiency of *Dnml1* abolishes mitochondrial autophagy and exacerbates HFrEF development^[243]^. Notably, alternative mitophagy also plays a role in HFrEF. TAC induced PO in mice results in a transient upregulation of ATG7-dependent mitophagy peaking at day 1 after TAC. However, ULK1/RAB9A dependent alternative mitophagy is also upregulated even stronger but with a delayed time course. TAC results in more severe cardiac dysfunction in *Ulk1* cardiac-specific KO mice. This can be circumvented with Tat-BECN1 treatment^[244]^. These results indicate a key role of alternative mitophagy in preserving cardiac function during PO when ATG7-dependent mitophagy is declined.

HFD consumption induces diastolic dysfunction, cardiac hypertrophy, and insulin resistance in mice^[196]^. The excessive accumulation of lipids and overload of free fatty acids in cardiomyocytes disrupt glucose metabolism and result in a shift in cardiac mitochondria substrate utilization toward increased fatty acid oxidation. This metabolic shift compromises oxidative phosphorylation, resulting in reduced ATP synthesis. Increased electron leakage from the electron transport chain promotes excessive ROS production, ultimately contributing to mitochondrial dysfunction^[245]^. To maintain cellular homeostasis, dysfunctional mitochondria can be removed through mitophagy. Therefore, induced mitophagy upon HFD consumption in mice is important for protection against diastolic dysfunction. Autophagy is activated by HFD and peaks at 6 weeks after which it is attenuated. However, mitophagy is increased after 3 weeks of HFD feeding and continues to increase beyond 2 months^[69]^. In this setting, cardiac-specific *Atg7* or *Prkn* deletion hampers mitophagy and exacerbates diastolic dysfunction in response to HFD. On the other hand, Tat-BECN1 administration counteracts HFD-induced cardiac dysfunction^[69]^. In the same mouse model, deletion of *Ulk1* abolishes mitophagy and exacerbates cardiac dysfunction. Impaired mitophagy and more severe cardiac dysfunction is also observed in RAB9A^S179A^ knock-in mice where alternative mitophagy is selectively suppressed. In contrast, increased mitophagy and protection from HFD-induced cardiac dysfunction are observed through cardiac-specific RAB9A overexpression. Furthermore, HFD induced activation of RAB9A alternative mitophagy is accompanied by upregulation of the transcription factor TFE3 (transcription factor binding to IGHM enhancer 3), crucial for transcriptional activation of mitophagy. Interestingly, DNM1L is involved in RAB9A mediated mitophagy during the chronic phase of HFD consumption^[72]^. Here, induction of alternative mitophagy is completely abolished in cardiac-specific conditional *dnm1l* KO mice resulting in exacerbated cardiac dysfunction, highlighting the importance of mitochondrial fission^[246]^. Taken together, these studies imply that induction of alternative mitophagy plays a crucial role in mitigating chronic phase HFD-induced cardiac dysfunction.

In contrast to the HFD mouse model described above where HFD induces mitophagy, modeling HFpEF in mice with HFD and L-NAME (2 hit model) leads to decreased fatty-acid oxidation resulting in impaired mitophagy^[247]^. Stimulation of mitophagy or induction of fatty acid oxidation improves the outcome of HFpEF in this model. This study highlights that fatty acid metabolism and mitochondria quality control through mitophagy are causally linked and underscores the importance of further investigations toward the underlying molecular mechanisms of HFpEF.

## Doxorubicin-induced cardiotoxicity

Anthracyclines, including doxorubicin, are widely used as chemotherapeutic agents in cancer treatment. However, their clinical application is limited by dose-dependent cardiotoxicity. Functionally, doxorubicin-induced cardiotoxicity (DIC) leads to reduced left ventricular ejection fraction, impaired diastolic relaxation, and maladaptive remodeling^[248]^. Morphological hallmarks include cardiomyocyte atrophy, vacuolization, interstitial fibrosis, and disruption of sarcomeres. Ultrastructural analyses show swollen and fragmented mitochondria with disrupted cristae, disorganized myofibrils, dilated sarcoplasmic reticulum, as well as accumulation of autophagic vacuoles^[249,250]^.

As an anticancer agent, doxorubicin works by disrupting mechanisms preventing double-stranded nuclear DNA breaks, thus inducing DNA damage. Additionally, doxorubicin increases production of ROS triggering cell death pathways and other stress responses that account for the drug’s efficacy in cancer treatment, while at the same time causing potentially life-threatening cardiac side effects^[251]^. Doxorubicin destabilizes the inner mitochondrial membrane, leading to disruption of ATP production, perturbation of mitochondrial dynamics, and release of cytochrome c into the cytosol, triggering apoptosis. Since cardiomyocytes do not regenerate, doxorubicin-induced cell death is particularly damaging in the heart.

Multiple lines of evidence point to a key role of autophagy and mitophagy in the etiology of doxorubicin-induced cardiotoxicity (DIC). Experiments using transgenic mouse models demonstrate that doxorubicin-induced cardiotoxicity (DIC) reflects not merely suppression or induction of autophagy, but rather maladaptive flux and impaired mitophagy, contributing to cardiac dysfunction.

### Role of autophagy in doxorubicin-induced cardiotoxicity

Doxorubicin can impair autophagic flux in hearts of rodent models, detected as increased MAP1LC3B-II and SQSTM1/p62 levels, without a further increase using lysosomal inhibitors, suggesting impaired autophagic flux^[252,253]^. In addition, doxorubicin treatment in RFP-GFP-MAP1LC3B reporter mice results in an accumulation of autolysosomes rather than autophagosomes. This indicates inhibition of autophagic flux downstream of autophagosome-lysosome fusion. Indeed, doxorubicin inhibits acidification of lysosomes and thus their function. In support of this, decreased levels of autophagy through *Becn1* haploinsufficiency relieve doxorubicin-induced decline in cardiac function in mice, while increased autophagy via BECN1 overexpression worsens this outcome^[253]^. Furthermore, *Uvrag*-deficient mice, that already suffer impaired autophagic flux due to hampered autophagosome-lysosome fusion, display exacerbated DIC^[254]^. In line with this, deficiency of RUBCN, the inhibitory partner of UVRAG, mitigates doxorubicin-induced impairment of autophagic flux^[255]^. Finally, suppression of autophagosome formation by overexpression of a dominant-negative variant of ATG5 ameliorates DIC in rats in the acute phase, but not in the long term^[256]^. These findings suggest that attenuated autophagy protects against cardiotoxicity in the early phase of DIC.

Several other genetic interventions that ameliorate DIC in mice function through the restoration of autophagic flux. For instance, deletion of TLR9 (toll-like receptor 9) exhibits cardioprotection by restoring autophagy and rescuing doxorubicin‐induced abnormal autophagy flux. Conversely, inhibition of autophagy in *tlr9* KO mice abolishes the cardioprotective effect^[257]^. Doxorubicin treatment leads to a reduction in plasma APOM (apolipoprotein M). This protein is a part of high-density lipoprotein (HDL) and APOM is inversely associated with mortality in human heart failure. Mice overexpressing APOM are protected from doxorubicin-induced cardiotoxicity through restoration of lysosomal function and enhanced autophagic flux involving the mTOR signaling pathway and TFEB^[258]^. These findings highlight that autophagy restoration is protective against DIC.

Doxorubicin-induced cardiac dysfunction is linked to ER stress in cardiomyocytes^[259]^. Notably, doxorubicin treatment induces ER-phagy in ER-phagy reporter mice and the expression level of ER-phagy receptors, including CCPG1 (cell cycle progression 1) is upregulated in doxorubicin treated cardiomyoblasts^[260]^. CCPG1 is a key protein facilitating ER-phagy under conditions of ER stress in mammalian cells^[261]^. In ER-phagy reporter mice with a suppression of CCPG1 expression, ER-phagy is abolished in response to doxorubicin treatment without affecting autophagic flux, while apoptosis and ER stress is increased. These results suggest that CCPG1-mediated ER-phagy is cardioprotective by mitigating doxorubicin-induced cardiotoxicity through ER-stress alleviation^[260,262]^.

### Role of mitophagy in doxorubicin-induced cardiotoxicity

Dysregulated mitophagy is linked to DIC phenotypes, and mitophagy activation is suggested as a potential therapeutic avenue for DIC. Interestingly, *parkin* KO mice display aggravated DIC. Furthermore, doxorubicin-activated TP53 can bind to PRKN and hinder its translocation from the cytosol to the mitochondrial membrane, thus blunting the mitophagy response. This leads to stalled mitophagy, and a failure to clear damaged mitochondria, associated with increased DIC^[152]^.

Receptor-mediated mitophagy is also implicated in DIC. Doxorubicin treatment induces excessive *Bnip3* expression, driving mitochondrial dysfunction and cardiomyocyte death. However, *bnip3* KO or expression of nonfunctional BNIP3 mutants protects against DIC^[263]^. Conversely, *Fundc1* deficiency in mice exacerbates mitochondrial injury and DIC^[264]^ while overexpression of FUNDC1 leads to improved cardiac functional outcomes^[265]^.

Taken together, studies in transgenic mouse models demonstrate that alterations in the level of autophagy and mitophagy impact on DIC in a context-dependent manner. Impaired autophagic flux and defective mitophagy amplify doxorubicin-induced injury, while balanced mitophagic clearance preserves cardiac function. Future strategies to mitigate DIC could therefore benefit from targeting selective enhancement of mitophagy and lysosomal capacity.

## Future perspectives/concluding remarks

Basal levels of autophagy and mitophagy as well as adaptive alterations in these pathways in response to stress contribute to preserve cardiovascular homeostasis. However, what determines the threshold for an appropriate and minimum effective level of autophagy or mitophagy in the different cells of the cardiovascular system to ensure proper function remains elusive. Moreover, parallel redundant mitophagy pathways appear to be at play in basal conditions and in certain disease settings in cardiac cells^[58,98,218]^. Perturbation of autophagy or mitophagy in the cardiovascular system in mice through global or cell-specific genetic modifications of core-autophagy machinery genes such as *Atg5* and *Atg7* or mitophagy linked genes such as *Prkn* and *Fundc1* offers valuable insights into the importance of these pathways, also in disease models. However, autophagy- or mitophagy-independent functions of these genes should be considered and these are still poorly characterized. Furthermore, as illustrated in Table 1, only a limited number of studies performed in mice undertake flux measurements. To assess autophagic flux, the use of inhibitors such as chloroquine or bafilomycin A_1_ is needed to discern whether a particular condition or intervention leads to an induction in autophagosome formation (autophagy induction) or a block in autophagosome degradation (autophagy inhibition). Indeed, assessment of autophagic or mitophagic flux *in vivo* is not an easy task without the use of genetically expressed pH-sensitive reporters, but is of outmost importance to decipher the underpinning roles of autophagy or mitophagy^[34]^.

The mechanisms governing the observed age-related decline in autophagy and mitophagy in the heart are unclear. Recently, elegant mapping of basal autophagy and mitophagy in the mouse brain during aging using pH-sensitive autophagy/mitophagy reporters demonstrates regional and cell subset-specific differences. Furthermore, in contrast to the prevailing conception, the results do not indicate a decline in mitophagy in the brain of aged mice^[65]^. If the same applies for the heart remains to be determined.

Interplay between different cell types also plays an important role in cardiovascular homeostasis. Long-lived cells such as cardiomyocytes are especially vulnerable to oxidative damage and must maintain efficient removal of harmful constituents. Interestingly, resident macrophages in the heart perform lysosomal degradation of dysfunctional mitochondria which are expelled from cardiomyocytes within large vesicles (exopheres) in a process termed heterophagy. The link between cardiomyocyte mitophagy and heterophagy is unknown but the release of exopheres from the cardiomyocytes depends on ATG7 and can be stimulated with rapamycin, indicating the involvement of the autophagy machinery^[266,267]^.

The level and alterations of autophagy and mitophagy in the human heart in health and disease are still mostly elusive. Plasma levels of ATG5 and BECN1 are lower in patients diagnosed with acute myocardial infarction compared to control subjects^[268]^. How this reflects alterations in cell or tissue-specific autophagy pathways remains unknown. Notably, detected altered plasma levels of SQSTM1 (increased) and ATG5 (reduced) in patients with thoracic aortic aneurysm correlated with aortic tissue levels of autophagy markers, indicating their potential as circulating biomarkers^[269]^. Interestingly, autophagic flux in humans can be measured using human whole blood samples treated with chloroquine. Here, autophagic flux is determined in peripheral blood mononuclear cells isolated from untreated or chloroquine treated fresh blood samples^[270]^. Further studies are needed to determine if autophagic flux in peripheral blood mononuclear cells correlates well with flux in other tissues and cardiovascular disease status. Assessment of autophagy and mitophagy in the human heart is limited by the availability of human heart tissue. Tissue biopsies from patients pre- and post-left ventricular assist device (LVAD) surgery can be used to assess alterations in autophagy markers in the heart following mechanical unloading by LVAD. A recent study indicates that LVAD mechanical unloading induces autophagic flux in patients with advanced heart failure, indicating involvement of autophagy in reverse remodeling of the heart^[271]^. While awaiting future innovative noninvasive assays that can reliably monitor autophagy and mitophagy in real-time in humans, engineered cardiac tissue constructs derived from human stem cells offer an alternative to animal studies^[272]^. Likewise, cultured human living myocardial slices from biopsies^[273]^ offer an opportunity to assess autophagic flux in human heart tissue using lysosomal inhibitors^[274]^. An improved understanding of physiological autophagy and mitophagy in tissue development, disease, and repair is a prerequisite for exploiting modulation of autophagy or mitophagy for therapeutic gain.

## Data Availability

Data sharing is not applicable to this article as no new data were created or analyzed in this work.

## Abbreviations

APOE: Apolipoprotein E
ATP: Adenosine triphosphate
ATG7: Autophagy related 7
ATG14: Autophagy related 14
BECN1: Beclin 1
BNIP3: BCL2 interacting protein 3
BNIP3L: BCL2 interacting protein 3 like
CHMP2B: Charged multivesicular body protein 2B
CRYAB: crystallin alpha B
DIC: Doxorubicin induced cardiotoxicity
DNM1L: Dynamin 1 like
ER: Endoplasmic reticulum
FBXO32: F-box protein 32
FICD: FIC domain protein adenylyltransferase
FUNDC1: FUN14 domain containing 1
FYCO1: FYVE and coiled-coil domain autophagy adaptor 1
HFD: High-fat diet
HF: Heart failure
HFpEF: Heart failure with preserved ejection fraction
HFrEF: Heart failure with reduced ejection fraction
I/R: Ischemia and reperfusion
KLF4: KLF transcription factor 4
KO: Knockout
LAD: Left anterior descending coronary artery
LAMP2: Lysosomal associated membrane protein 2
LDLR: Low density lipoprotein receptor
LIRs: LC3 interacting regions
L-NAME: N[w]-nitro-l-arginine methyl ester
LVEF: Left ventricular ejection fraction
MAP1LC3B: Microtubule associated protein 1 light chain 3 beta
MFN1: Mitofusin 1
MFN2: Mitofusin 2
miR: Micro RNA
MST1: Macrophage stimulating 1
MTORC1: Mechanistic target of rapamycin complex 1
NO: Nitric oxide
OxLDL: Oxidized low density lipoproteins
PDGF: Platelet-derived growth factor
PGAM5: PGAM family member 5, mitochondrial serine/threonine protein phosphatase
PINK1: PTEN induced putative kinase 1
PO: Pressure overload
PRKAA2: Protein kinase AMP-activated catalytic subunit alpha 2
PRKN: Parkin RBR E3 ubiquitin protein ligase
PtdIns3K-C1: Class III phosphatidylinositol 3-kinase complex I
RAB9A: RAB9A, member RAS oncogene family
RHEB: Ras homolog, mTORC1 binding
ROS: Reactive oxygen species
RPTOR: The regulatory associated protein of MTOR complex 1
RUBCN: Rubicon autophagy regulator
SARs: Selective autophagy receptors
SLC39A7: solute carrier family 39 member 7
SQSTM1: Sequestosome 1
SYT7: Synaptotagmin 7
TAC: Transverse aortic constriction
TFEB: Transcription factor EB
TFRC: Transferrin receptor
ULK1: Unc-51 like autophagy activating kinase 1
UVRAG: UV radiation resistance associated
VECs: Vascular endothelial cells
VSMCs: Vascular smooth muscle cells
VWF: Von Willebrand factor
